# Genotypic and Phenotypic Diversity of Herpes Simplex Virus 2 within the Infected Neonatal Population

**DOI:** 10.1128/mSphere.00590-18

**Published:** 2019-02-27

**Authors:** Lisa N. Akhtar, Christopher D. Bowen, Daniel W. Renner, Utsav Pandey, Ashley N. Della Fera, David W. Kimberlin, Mark N. Prichard, Richard J. Whitley, Matthew D. Weitzman, Moriah L. Szpara

**Affiliations:** a Department of Pediatrics, Division of Infectious Diseases, Children’s Hospital of Philadelphia, Philadelphia, Pennsylvania, USA; b Department of Biochemistry and Molecular Biology, Center for Infectious Disease Dynamics, The Huck Institutes of the Life Sciences, Pennsylvania State University, State College, Pennsylvania, USA; c Department of Pathology and Laboratory Medicine, Division of Protective Immunity and Division of Cancer Pathobiology, Children’s Hospital of Philadelphia, Philadelphia, Pennsylvania, USA; d Department of Pediatrics, Division of Infectious Diseases, University of Alabama at Birmingham, Birmingham, Alabama, USA; e University of Pennsylvania Perelman School of Medicine, Philadelphia, Pennsylvania, USA; University of Arizona

**Keywords:** comparative genomics, herpes simplex virus, human herpesvirus 2, minor variants, neonatal, viral spread

## Abstract

More than 14,000 neonates are infected with herpes simplex virus (HSV) annually. Approximately half display manifestations limited to the skin, eyes, or mouth (SEM disease). The rest develop invasive infections that spread to the central nervous system (CNS disease or encephalitis) or throughout the infected neonate (disseminated disease). Invasive HSV disease is associated with significant morbidity and mortality, but the viral and host factors that predispose neonates to these forms are unknown. To define viral diversity within the infected neonatal population, we evaluated 10 HSV-2 isolates from newborns with a range of clinical presentations. To assess viral fitness independently of host immune factors, we measured viral growth characteristics in cultured cells and found diverse *in vitro* phenotypes. Isolates from neonates with CNS disease were associated with larger plaque size and enhanced spread, with the isolates from cerebrospinal fluid (CSF) exhibiting the most robust growth. We sequenced complete viral genomes of all 10 neonatal viruses, providing new insights into HSV-2 genomic diversity in this clinical setting. We found extensive interhost and intrahost genomic diversity throughout the viral genome, including amino acid differences in more than 90% of the viral proteome. The genes encoding glycoprotein G (gG; US4), glycoprotein I (gI; US7), and glycoprotein K (gK; UL53) and viral proteins UL8, UL20, UL24, and US2 contained variants that were found in association with CNS isolates. Many of these viral proteins are known to contribute to cell spread and neurovirulence in mouse models of CNS disease. This report represents the first application of comparative pathogen genomics to neonatal HSV disease.

**IMPORTANCE** Herpes simplex virus (HSV) causes invasive disease in half of infected neonates, resulting in significant mortality and permanent cognitive morbidity. The factors that contribute to invasive disease are not understood. This study revealed diversity among HSV isolates from infected neonates and detected the first associations between viral genetic variations and clinical disease manifestations. We found that viruses isolated from newborns with encephalitis showed enhanced spread in culture. These viruses contained protein-coding variations not found in viruses causing noninvasive disease. Many of these variations were found in proteins known to impact neurovirulence and viral spread between cells. This work advances our understanding of HSV diversity in the neonatal population and how it may impact disease outcome.

**Podcast**: A podcast concerning this article is available.

## Abstract (precis)

Herpes simplex virus (HSV) causes invasive disease in half of infected neonates, resulting in significant mortality and permanent cognitive morbidity. The factors that contribute to invasive disease are not understood. This study revealed diversity among HSV isolates from infected neonates and detected the first associations between viral genetic variations and clinical disease manifestations. We found that viruses isolated from newborns with encephalitis showed enhanced spread in culture. These viruses contained protein-coding variations not found in viruses causing noninvasive disease. Many of these variations were found in proteins known to impact neurovirulence and viral spread between cells. This work advances our understanding of HSV diversity in the neonatal population and how it may impact disease outcome.

## INTRODUCTION

Each year, an estimated 10,000 neonates are infected with herpes simplex virus 2 (HSV-2), and 4,000 are infected with HSV-1, worldwide (1). Infants are typically infected at the time of birth by maternal genital shedding of HSV, most often by mothers who are not aware of their infection (2–4). The recent increase in genital HSV-1 incidence among women of childbearing age, particularly in developed nations, suggests that the burden of neonatal infection will continue to rise (1, 5). While some infected infants exhibit only superficial infection limited to the skin, eyes, or mouth (SEM disease; 45%), about half develop invasive systemic infections (disseminated [DISS] disease; 25%) or infections of the central nervous system (CNS disease; 30%) associated with significant morbidity and mortality (6, 7). Currently, administration of the antiviral medication acyclovir is the standard therapy for all forms of neonatal HSV disease. Although this intervention has reduced mortality due to invasive disease, most survivors of invasive disease are left with permanent neurodevelopmental deficits (8, 9).

The factors that predispose a neonate to invasive HSV infection are not entirely known. Recent studies have found that some adults and children outside the neonatal period who experience HSV infection of the brain have a host genetic defect within the Toll-like receptor-3 (TLR3) pathway (10, 11). Outside the neonatal period, HSV encephalitis is rare, as are host defects in the TLR3 pathway. In contrast, half of HSV-infected neonates experience invasive CNS or disseminated disease, making it less likely that host genetic defects alone could account for all of the observed cases of invasive infection in neonates. Prior clinical data on mother-to-infant transmission of HSV indicate that most cases of neonatal disease, including invasive forms of disease, result from newly acquired or primary HSV infection prior to the development of maternal antibody production (2–4, 12). This suggests a window of opportunity where the contributions of viral genetic variation to the progression of invasive infection and disease may be greater in neonates than in adults.

Prior studies have identified viral genetic factors that influence virulence or disease for reoviruses, influenza virus, HIV, and other viruses (13–18). In contrast to these RNA viruses, HSV was presumed to have lower genetic diversity and potential for variation in virulence, due to its relatively stable DNA genome and long coevolutionary history with humans (19). The assumption of limited HSV heterogeneity was supported by early studies that utilized low-resolution restriction fragment length polymorphism (RFLP) or single-gene analyses to compare multiple HSV isolates (20–22). However, Rosenthal and colleagues used RFLP and PCR analysis of a single locus to demonstrate that a heterogeneous HSV population can exist in an invasive neonatal infection and provided proof of principle that natural genetic variation can impact neurovirulence (23, 24). More recently, advances in high-throughput sequencing (HTSeq) have enabled a reevaluation of herpesvirus genome-wide variation, which suggests that herpesviruses harbor extensive diversity both between strains or individuals (interhost variation) as well as within a single individual (intrahost variation) (25–27). These minor genetic variants may become clinically important if a variant within the viral population becomes the new dominant allele or genotype as a result of a bottleneck at transmission, entry into a new body compartment, or selective pressure such as that represented by antiviral therapy (28, 29).

Several recent examples have demonstrated the potential for gaining new insights by applying HTSeq approaches to herpesvirus infections in a clinical setting. In HTSeq studies of congenital infection by the beta-herpesvirus human cytomegalovirus (HCMV), Renzette et al. found evidence for heterogeneous viral populations both within and between hosts (30–35). The levels of diversity observed in congenital HCMV infections far exceeded those observed in adult infections (36–38). HTSeq-based examination of vaccine-associated rashes due to the alphaherpesvirus varicella zoster virus (VZV) demonstrated that adult skin vesicles contain a subset of the viral population introduced during vaccination and revealed at least 11 VZV genomic loci that were linked to rash formation (26). Recent HTSeq comparisons of adult genital HSV-2 isolates revealed the first evidence of a single individual shedding two distinct strains (39), demonstrated changes in the viral genome over time in a recently infected host (40), and provided the first evidence of ancient recombination between HSV-1 and HSV-2 (41, 42). To date, however, there has been no evaluation of genome-wide variation in neonatal HSV isolates to determine the levels of diversity in this population or the potential impact(s) of viral genetic variants on disease.

Until recently, several technical barriers prevented thorough assessment of neonatal HSV genomes. A key constraint on studies of neonatal disease has been the limited availability of cultured, minimally passaged viral samples that are associated with clinical information and have also been maintained in a low-passage-number state that is appropriate for sequencing and further experimental studies. Historically, viral culture was part of the HSV diagnostic workflow, but this has been superseded in clinical laboratory settings by the speed and sensitivity of viral detection by PCR (6, 43, 44). This change limits neonatal HSV sample availability for *in vitro* and animal model studies. In addition, many previously archived neonatal HSV isolates have been subjected to extensive passage, allowing them to acquire mutations that enhance viral growth in culture (23, 24). Additional challenges for HTSeq approaches to analysis of neonatal HSV include the large size (∼152 kb) of the viral genome, its high (∼70%) G+C content, and the large number of variable-number tandem repeats in the viral genome (>240 minisatellite/microsatellite repeats and >660 homopolymers of ≥6 bp) (27, 45). Therefore, many studies of HSV diversity (21, 46–49) or of the effect of HSV genetic variation on disease (50–52) have relied on low-resolution RFLP or single-gene PCR analyses due to the speed and ease of analysis in comparison to whole-genome approaches (53–58). To overcome these challenges, we combined our expertise in HSV comparative genomics and phenotypic analysis (53, 54, 58, 59) with a unique resource of low-passage-number, well-annotated neonatal specimens (8, 9, 60).

Here we analyzed a set of 10 low-passage-number clinical HSV-2 isolates collected from neonates with HSV infection who had been enrolled in one of two clinical studies that spanned 3 decades of patient enrollment (1981 to 2008) (8, 9, 60). These samples represented a wide range of clinical manifestations, including SEM, CNS, and disseminated disease, and each sample was associated with deidentified clinical information. We defined the level of diversity in this population using comparative genomics and an array of cell-based phenotypic assays. We found that HSV-2 isolates displayed diverse *in vitro* phenotypes as well as extensive interhost and intrahost diversity distributed throughout the HSV-2 genome. Finally, we found coding variations in several HSV-2 proteins associated with CNS disease. This report represents the first-ever application of comparative pathogen genomics to neonatal HSV disease and provides a basis for further exploration of genotype-phenotype links in this clinically vulnerable patient population.

## RESULTS

### Neonatal HSV-2 samples represent a diverse clinical population.

We utilized samples collected from 10 HSV-2-infected neonates enrolled by the National Institute of Allergy and Infectious Diseases Collaborative Antiviral Study Group (CASG) for clinical trials between 1981 and 2008 (8, 9, 60). These infants encompassed a range of clinical disease manifestations (see Table 1), with about half experiencing invasive CNS disease (5 patients) or disseminated (DISS) disease with CNS involvement (2 patients) and the remainder experiencing noninvasive SEM disease (3 patients). Extensive clinical information was available for each patient, including long-term neurocognitive and motor outcomes (Table 1). This population was also diverse with respect to sex, race, gestational age, and enrollment center (Table 1; enrollment center data not shown). All samples were collected at the time of diagnosis, prior to initiation of acyclovir therapy. Each isolate was cultured once as part of the diagnostic process, with expansion performed only for the experiments described here. Although the sample size was constrained by the rarity of neonatal HSV infection and availability of appropriately maintained isolates, our group is similar in size to groups used for prior HTSeq comparisons of congenital HCMV samples (30–35) and represents the largest group of neonatal HSV samples ever subjected to comparative genomic and phenotypic analysis.

**TABLE 1:** Clinical characteristics associated with HSV-2 isolates from 10 patients

Clinical isolate^*a*^	Clinical disease(s) at diagnosis	Sample source	Morbidity score		Patient age at disease onset (days)	Gestational age at time of birth (wks)	Patient sex and race^*b*^
			Mental	Motor			
CNS11	CNS	CSF	4	4	12	37	M, W
DISS14	DISS + CNS	CSF	2	2	7	39	M, W
CNS03	CNS	Skin	4	4	17	37	M, W
CNS15	CNS	Skin	3	4	19	36	M, W
CNS17	CNS	Skin	4	4	17	40	M, W
DISS29	DISS + CNS	Skin	3	3	5	38	F, B
CNS12	CNS	Skin	4	4	16	41	F, W
SEM02	SEM	Skin	1	1	5	38	F, W
SEM13	SEM	Skin	4	4	11	27	F, B
SEM18	SEM	Skin	2	2	17	37	F, W

a Clinical isolate order based on data in Fig. 1.

b F, female; M, male; B, black; W, white.

### Neonatal HSV-2 isolates have different levels of fitness in culture.

To determine whether the viruses isolated from this neonatal population (Table 1) were intrinsically different, we assessed viral growth in culture, which provides a consistent environment that is independent of host genetic variation. To minimize the impact of immune pressure, we selected Vero monkey kidney cells, which lack an interferon response (61, 62). Each viral isolate was applied to a confluent monolayer of cells *in vitro* and allowed to form plaques for 100 h (Fig. 1A). The average plaque sizes differed between isolates, with the plaques seen with 6 of the 10 isolates being statistically significantly larger (indicated in green) than those seen with the other 4 (indicated in black; Fig. 1B) (one-way analysis of variance [ANOVA] with Holm-Sidak’s multiple-comparison test; *P* < 0.05). The average plaque size of the previously described low-passage-number, adult HSV-2 isolate SD90e is shown for comparison (63). The largest plaque sizes were observed in the two viruses isolated directly from the cerebrospinal fluid (CSF; isolates CNS11 and DISS14; Fig. 1B); these two isolates were not statistically significantly different from one another in size but were significantly larger than any of those isolated from the skin (one-way ANOVA with Holm-Sidak’s multiple-comparison test, *P* < 0.05). Plaque size was assessed at each passage in culture and remained constant from the time the isolates were received in our laboratory (passage 2) through their genetic and phenotypic analysis (passage 4). The variances in plaque sizes produced by a given isolate were not statistically significantly different between isolates (Fig. 1B). The differences in average plaque sizes between isolates suggested that the HSV-2 populations found in each neonatal isolate were indeed intrinsically different.

**FIG 1:**
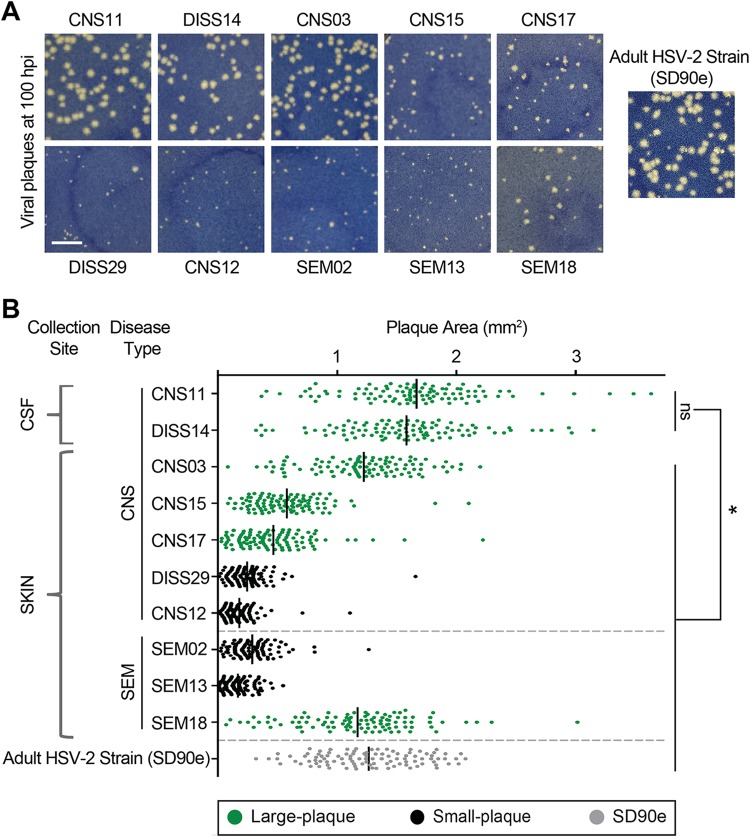
Neonatal HSV-2 isolates generate plaques of different sizes in culture. (A) Plaques representative of those seen after virus incubation on Vero cells for 100 h are shown. Previously described low-passage-number adult HSV-2 strain SD90e (63) is shown for comparison. Scale bar = 5 mm. (B) Quantification of plaque area on Vero cells. Dots represent 100 individually measured plaques, and black bars represent means. Each green isolate (Large-plaque) is statistically significantly larger than each black isolate (Small-plaque). Black isolates are not statistically significantly different from one another. Additionally, each CSF-derived isolate is statistically significantly larger than all other isolates shown. Collection site and disease type are indicated on the left (see Table 1 for details). For all statistics, *P* values are <0.05 by one-way ANOVA followed by Holm-Sidak’s multiple-comparison test.

### Entry kinetics, DNA replication, protein expression, and virus production do not account for differences in plaque size.

**FIG 2:**
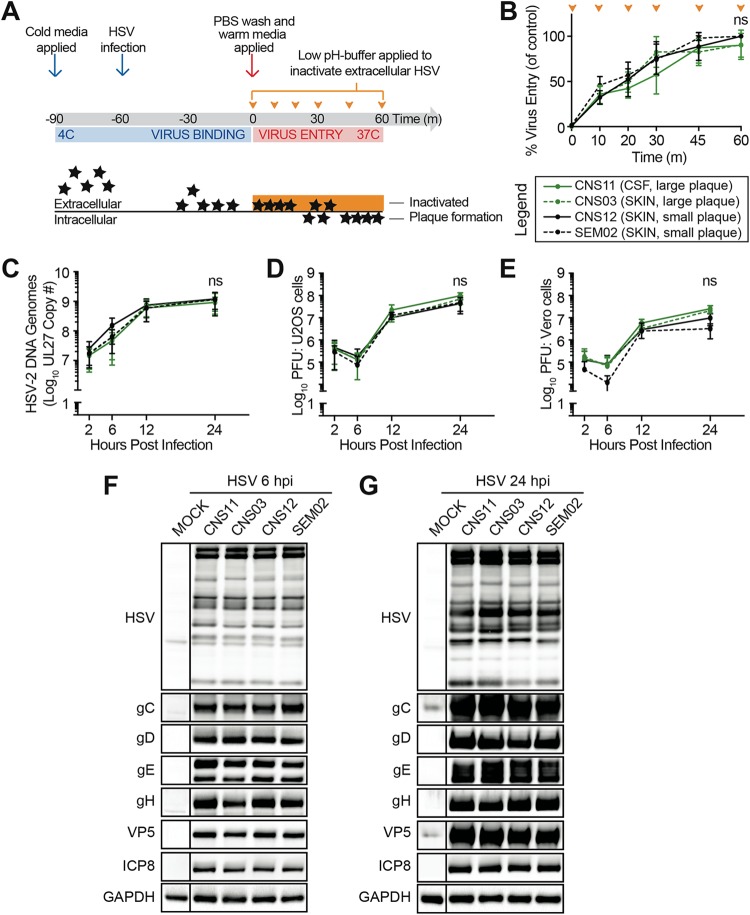
Increased plaque size in culture is not determined by viral entry, DNA replication, protein expression, or infectious virus production. Viral growth characteristics were compared for representative neonatal isolates, including large-plaque formers (green) and small-plaque formers (black). (A and B) Viral entry kinetics. (A) Viral isolates were applied to Vero cell monolayers at 4°C for 1 h to allow virus binding and were then moved to 37°C to allow virus entry. Extracellular virus was inactivated by a low-pH buffer at the times indicated (orange arrowheads). Cell monolayers were washed and overlaid with methylcellulose. Plaques were scored after 100 h of incubation. PBS, phosphate-buffered saline. (B) Viral entry was quantified as the fraction of plaques formed following citrate buffer application, where 100% represents the number of plaques formed on a monolayer not treated with citrate buffer (control). These data represent results from three independent experiments. Two-way ANOVA followed by Tukey’s multiple-comparison test was applied. (C to E) Single-cycle viral replication kinetics. Vero cell monolayers were infected at MOI = 5 and incubated in the presence of 0.1% human serum. Cell monolayers were harvested at the time points indicated. (C) The quantity of viral genomes present was evaluated by qPCR for UL27. (D and E) Infectious virion production (titer) was evaluated by plaque formation on U2OS (D) or Vero (E) cells. These data represent results from three independent experiments. Two-way ANOVA followed by Tukey’s multiple-comparison test was applied. (F and G) Protein production. Vero cell monolayers were infected at MOI = 5 for 6 h (F) or 24 h (G). Whole-cell lysates were subjected to immunoblot analysis with the following antibodies: gC (UL44), gD (US6), gE (US8), gH (UL22), four virion glycoproteins; VP5 (UL19), capsid protein; ICP8 (UL29), viral single-strand DNA-binding protein; HSV, viral antibody against whole HSV-1; GAPDH, cellular glyceraldehyde-3 phosphate dehydrogenase as a loading control.

Plaque formation is a complex endpoint that involves the ability of the virus to enter the cell, replicate its double-stranded DNA genome, produce viral proteins, and assemble new virions that then spread to adjacent cells. Therefore, we explored whether the differences in plaque formation observed in Vero cells reflected inherent differences in the abilities of isolates to complete each of these stages of the viral life cycle. For these comparisons, two large-plaque-forming isolates (CNS11 and CNS03) were compared to two small-plaque-forming isolates (CNS12 and SEM02). First, we compared the rates of cell entry seen with the isolates. Virus was applied to chilled cells, followed by warming to synchronize cell entry (Fig. 2A). A low-pH solution was applied at various points over the first hour of cell entry to inactivate any virus that had not yet entered a cell, and plaque formation was then allowed to proceed for 100 h. We found no difference between these four representative viral isolates in their rates of cell entry (Fig. 2B), suggesting that the large plaques did not result from increased rates of virus entry into cells.

We next infected Vero cells at a high multiplicity of infection (MOI = 5) to compare the outcomes of a single round of viral replication. We found that the four isolates produced similar numbers of genome copies (as measured by quantitative PCR [qPCR] for the gB gene; see Materials and Methods for details) (Fig. 2C), indicating that differences in viral DNA replication did not influence plaque size. We quantified the production of infectious virus by counting plaque-forming-units (PFU) on Vero cell monolayers as well as on cells of the highly permissive U2OS human bone osteosarcoma epithelial cell line (64). No differences in virus production were noted between the four isolates in quantification on either cell type (Fig. 2D and E). U2OS cells lack innate sensing of viral infection through the STING pathway (65) and can even support the growth of highly defective HSV isolates that lack ICP0 function (64). All isolates formed large plaques on the highly permissive U2OS cell monolayers (see Fig. S1 in the supplemental material), allowing us to rule out the possibility that very small foci of infection were missed during determination of the titers of the small-plaque-forming isolates on Vero cells (Fig. 2D and E). Finally, we compared viral protein production levels for these isolates and found no differences in the expression levels of a panel of HSV-2 viral proteins at either early (6 h postinfection [hpi]) or late (24 hpi) time points of a single round of high-MOI infection (Fig. 2F and G; see legend for the list of proteins). Taken together, these results suggested that the large-plaque-forming and small-plaque-forming neonatal HSV-2 isolates did not differ significantly in viral entry, DNA replication, infectious virus production, or protein production over a single round of infection.

10.1128/mSphere.00590-18.2 FIG S1 U2OS cells support large-plaque formation by all isolates. Neonatal viruses were allowed to incubate for 100 h on Vero or U2OS cells, and representative plaques are shown. All neonatal isolates were capable of forming large plaques on U2OS cells, which lack innate sensing of viral infection through the STING pathway (65). Download FIG S1, TIF file, 2.9 MB. Copyright © 2019 Akhtar et al. 2019 Akhtar et al. This content is distributed under the terms of the Creative Commons Attribution 4.0 International license.

### Large-plaque-forming isolates exhibit enhanced cell-to-cell spread.

**FIG 3:**
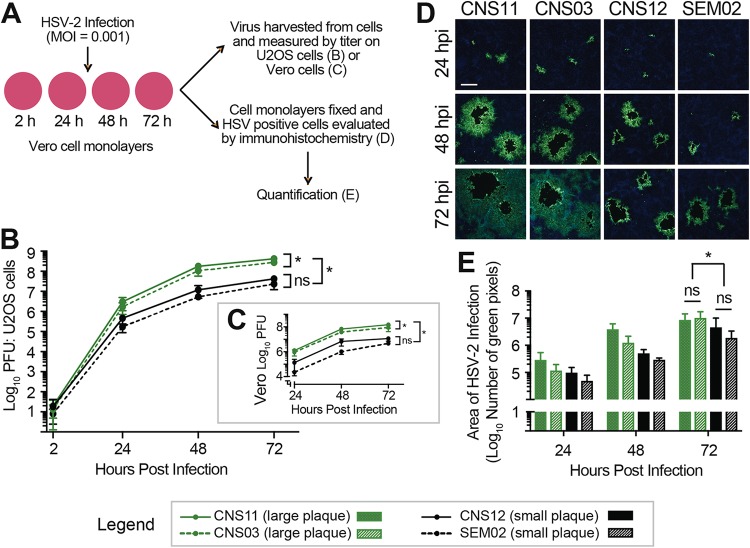
Enhanced viral cell-to-cell spread contributes to increased plaque size in culture. (A) The rates of viral spread in Vero cells were compared for representative neonatal isolates, including large-plaque formers (green) and small-plaque formers (black). Vero cell monolayers were infected at MOI = 0.001 in the presence of 0.1% human serum, which was replenished every 24 h. (B and C) Samples were harvested at each time point, and viral titers were evaluated by plaque formation on U2OS cells (B) or Vero cells (C). These data represent results from three independent experiments. Two-way ANOVA was performed followed by Tukey’s multiple-comparison test. *, *P* < 0.0001 at 72 h. (D) In parallel experiments, HSV-positive cells (green) were evaluated by immunofluorescence. Cell nuclei are counterstained with DAPI (blue). Scale bar = 200 μm. Images are representative of results from three independent experiments. Images of the entire 10-mm coverslips were then captured and stitched to create a composite image (see Fig. S2). (E) The total number of immunofluorescent (green) pixels was quantified for each coverslip. Two-way ANOVA was performed followed by Tukey’s multiple-comparison test. *, *P* < 0.05 at 72 h.

We next assessed the ability of representative large-plaque-forming and small-plaque-forming isolates to spread from cell to cell. Vero cell monolayers were infected at a low MOI (MOI = 0.001) in order to assess differences over multiple rounds of viral replication and spread throughout the cell monolayer (Fig. 3A). The contribution of indirect cell-free spread was minimized by inclusion of 0.1% human serum in the media and by changing the media every 24 h to remove released virus and refresh serum levels. Over a 72-h time course, cell monolayers were assessed to measure the extent of cell-to-cell spread, either by harvesting and determining the titers of PFU production (Fig. 3B and C) or by fixing infected cell monolayers and evaluating the distribution of virus by immunofluorescence (Fig. 3D and E; see also Fig. S2). Viral titers recovered from harvested cells were similar at 2 hpi, confirming that equivalent amounts of each virus were present following the initial infection (Fig. 3B). By 72 hpi, after multiple rounds of replication and spread, large-plaque-forming isolates CNS11 and CNS03 had achieved viral titers significantly greater than those measured for small-plaque-forming isolates CNS12 and SEM02 (Fig. 3B and C; two-way ANOVA followed by Tukey’s multiple-comparison test, *P* < 0.0001 at 72 h). Isolate CNS11, which was obtained directly from the CSF, produced titers statistically greater than those produced by the other three isolates (Fig. 3B and C).

10.1128/mSphere.00590-18.3 FIG S2 Cell-to-cell spread is enhanced in certain neonatal HSV-2 isolates. Confluent Vero cell monolayers were infected at MOI = 0.001 for the time points indicated, in the presence of 0.1% human serum. HSV-positive cells (green) were evaluated at each time point by immunofluorescence. Cell nuclei were counterstained with DAPI (blue). Serial 10× images were obtained on an EVOS FL Auto Imaging system and stitched together to create an image of the entire experimental coverslip. Scale bar = 1mm. These images were quantified in Fig. 3E. Download FIG S2, PDF file, 0.7 MB. Copyright © 2019 Akhtar et al. 2019 Akhtar et al. This content is distributed under the terms of the Creative Commons Attribution 4.0 International license.

We also directly evaluated cell-to-cell spread by immunostaining and quantifying the distribution of infected cells around each infectious focus. Infected Vero cell monolayers were fixed at 24, 48, and 72 hpi and were subjected to fluorescent immunocytochemistry analysis using an antibody directed against total HSV (Fig. 3D; see also Fig. S2). The region of HSV-positive cells surrounding a single initial infection was greater for the large-plaque-forming neonatal HSV-2 isolates at 24 hpi and had increased by 48 and 72 hpi. The central cytolytic clearings seen in the monolayers infected by CNS11 or CNS03 were approximately 2-fold greater than those in the monolayers infected by CNS12 or SEM02, reflecting the average 2-fold increase in plaque size observed in methylene-blue-stained monolayers shown in Fig. 1. However, the region of infected cells surrounding the central cytolytic clearing was dramatically larger for CNS11 and CNS03 than for CNS12 and SEM02, suggesting that large-plaque-forming neonatal HSV-2 isolates show greater spread from cell to cell than would have been predicted by measuring plaque size alone. To quantify this increase in the area of infected cells after a low-MOI infection, each coverslip was imaged (Fig. S2) and the total number of immunofluorescent pixels was quantified (Fig. 3E). By 72 hpi, the area of HSV-infected cells was statistically greater for large-plaque-forming isolates CNS11 and CNS03 than for small-plaque-forming isolates CNS12 and SEM02 (two-way ANOVA followed by Tukey’s multiple-comparison test, *P* < 0.05 at 72 h). Together, these data indicated that large-plaque-forming isolates shared an enhanced ability to spread cell to cell through culture in comparison to small-plaque-forming isolates.

### Comparative genomics reveals genetic diversity in neonatal HSV-2 isolates.

**FIG 4:**
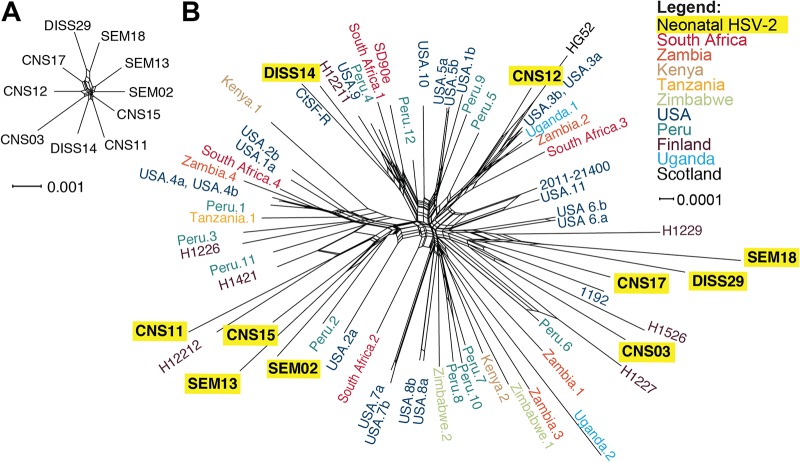
Neonatal HSV-2 genomes are genetically distinct from one another and encompass a broad range of known HSV-2 genetic diversity. A phylogenetic network constructed among neonatal HSV-2 genomes (A) or neonatal and adult HSV-2 genomes (B) reveals the wide genetic distribution of these unrelated isolates. HSV-2 genomes have been previously noted to lack geographic separation into clades (41, 42, 81). The network was created using SplitsTree4, from a MAFFT trimmed genome alignment. See Fig. S3 for a comparison tree constructed using a neighbor-joining (NJ) algorithm. See Table S1 for a complete list of accession numbers, geographic origins, and references for all 58 adult HSV-2 strains.

The differences identified in cell-to-cell spread between neonatal isolates in culture indicated the existence of intrinsic differences between these viruses. To reveal how genetic variation may contribute to viral phenotypes in culture and, ultimately, to clinical disease manifestations, we sequenced the complete viral genome of all 10 neonatal HSV-2 isolates (Table 2). For each isolate, we sequenced purified viral nucleocapsid DNA and assembled a consensus genome, which represented the most common genotype at each nucleotide locus in the viral population. The clinical trials utilized in this study enrolled HSV-infected infants from multiple sites across the United States (8, 9, 60). Therefore, we first assessed the overall degree of relatedness between these viral genomes to understand whether any similarities in viral or geographic origins might have contributed to *in vitro* or clinical phenotype patterns. In light of the known potential for recombination in the phylogenetic history of HSV (42, 49, 53, 66), we used a graph-based network to investigate the phylogenetic relationships among these isolates. We found similar degrees of divergence among all 10 neonatal HSV-2 consensus genomes (Fig. 4A). We then compared these 10 neonatal HSV-2 genomes to all available HSV-2 genomes in GenBank (all of which are derived from adults; see Table S1 for HSV-2 GenBank accession numbers and references) to discern any geographic or other clustering. The neonatal isolates did not segregate into any exclusive groupings but instead intermingled among the nonneonatal isolates, with deep branches between each pair of isolates (Fig. 4B). These findings were corroborated using an alternative phylogenetic clustering algorithm as well (Fig. S3). These data suggested that similarities in viral genetic origin were not responsible for determining the cellular or clinical outcomes of neonatal HSV-2 infection.

**TABLE 2:** Genome sequencing statistics for neonatal HSV-2 strains

Clinical isolate^*a*^	Avg coverage	No. of raw sequence reads	No. of reads used for assembly^*b*^	% viral reads	% of reads with depth >100	GenBank accession no.
CNS11	6,081×	3.5 million	2.8 million	79	96	MK105996
DISS14	6,267×	3.8 million	3.0 million	77	97	MK106000
CNS03	6,269×	3.9 million	3.1 million	78	96	MK105995
CNS15	7,486×	6.1 million	4.4 million	73	97	MK105998
CNS17	5,059×	3.1 million	2.3 million	73	96	MK105999
DISS29	2,588×	1.4 million	1.1 million	78	99	MK106001
CNS12	5,839×	3.9 million	2.8 million	73	96	MK105997
SEM02	6,455×	4.8 million	3.7 million	77	95	MK106002
SEM13	5,291×	3.4 million	2.4 million	72	89	MK106003
SEM18	7,514×	16.9 million	12.2 million	72	98	MK106004

a The clinical isolate order is based on data in Fig. 1.

b Numbers reflect the count of sequence read 1 of paired-end reads.

10.1128/mSphere.00590-18.4 FIG S3 Phylogenetic clustering demonstrated that neonatal HSV-2 genomes are genetically distinct from one another and intermingle within the previously known range of HSV-2 genetic diversity. A neighbor-joining (NJ) tree network constructed using 10 neonatal and 58 adult HSV-2 genomes revealed the wide genetic distribution of the neonatal isolates. The NJ tree (Jukes-Cantor; 1,000 bootstraps) was created in MEGA from a MAFFT trimmed genome alignment. Bootstrap values of ≥70 are shown here. See Fig. 4 for a network graph comparison to this tree. Table S1 contains a complete list of accession numbers, geographic origins, and references for all of the adult HSV-2 strains. Download FIG S3, TIF file, 0.8 MB. Copyright © 2019 Akhtar et al. 2019 Akhtar et al. This content is distributed under the terms of the Creative Commons Attribution 4.0 International license.

10.1128/mSphere.00590-18.7 TABLE S1 Accession numbers, geographic origins, and references for all of the adult HSV-2 genomes (58 in total) used for comparative genomic analyses. Download Table S1, PDF file, 0.1 MB. Copyright © 2019 Akhtar et al. 2019 Akhtar et al. This content is distributed under the terms of the Creative Commons Attribution 4.0 International license.

### The overall protein-coding diversity in neonatal HSV-2 isolates is similar to that observed in adult HSV-2 strains.

**FIG 5:**
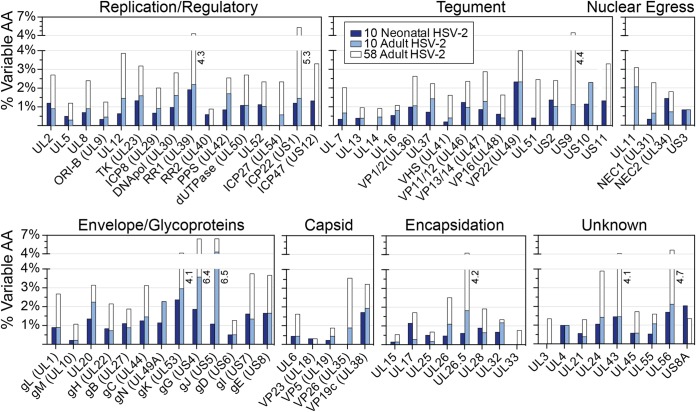
Neonatal HSV-2 proteins harbor consensus-level amino acid (AA) differences at a frequency similar to that seen between adult HSV-2 isolates. HSV-2 proteins are grouped by function, and the percentages of variable amino acids in each protein (representing the number of amino acid differences divided by protein length) are plotted for the 10 neonatal isolates (dark blue), for 10 representative adult HSV-2 isolates (light blue), and for all 58 adult HSV-2 genomes annotated in GenBank (clear outlines behind light blue bars). All adult HSV-2 genomes used for this comparison are listed in Table S1. See Table S2 for a numerical summary of nucleotide and AA differences observed in each set of neonatal or adult HSV-2 genomes.

We next asked whether overt defects in any single HSV-2 protein might be associated with clinical or *in vitro* spread phenotypes. In examining the coding potential of all 10 neonatal HSV-2 isolates, we found no protein deletions or truncations encoded by any of these viral genomes. These comparisons revealed a total of 784 nucleotide differences in 71 genes (see Table S2) that resulted in 342 nonsynonymous amino acid differences in 65 proteins. This led to an average coding variation level of 1% (range, 0.0% to 2.6%). This variation was spread widely throughout the HSV-2 genome, without any concentration in a particular genomic region or category of protein function (Fig. 5). The level of coding diversity in this set of neonatal isolates was similar to that detected in a comparable set of 10 adult HSV-2 isolates (Fig. 5; see also Table S2). We also compared these neonatal genomes to the full set of 58 annotated adult HSV-2 genomes from GenBank (listed in Table S1). As expected from the differences in sample size, there was more overall diversity found across the 58 adult HSV-2 genomes than in the 10 neonatal genomes. A comparison of the dN/dS ratios in the neonatal versus the adult HSV-2 genomes revealed similar trends, with a few outliers visible on each axis; e.g., UL38 and US8A had a higher dN/dS ratio in neonatal isolates than in adult isolates (see Table S2 and Fig. S4 for full comparisons). These data indicated that at the consensus level, the neonatal HSV-2 isolates displayed substantial interhost coding diversity spread throughout the genome but did not possess strikingly more diversity or an excess of genetic drift compared to the adult isolates.

10.1128/mSphere.00590-18.5 FIG S4 Ratio of nonsynonymous to synonymous coding variations in neonatal HSV-2 versus adult HSV-2 strains. The ratios of nonsynonymous (dN) to synonymous (dS) coding variations were plotted for each HSV-2 protein. The *x*-axis value represents the average dN/dS ratio for each protein in 58 adult HSV-2 strains, while the *y*-axis value represents the average dN/dS ratio in 10 neonatal isolates. Proteins with a difference in average dN/dS ratios of ≥1 in neonatal versus adult HSV-2 genomes are labeled (green indicates a higher average dN/dS ratio in neonatal HSV-2 genomes; red indicates a higher dN/dS ratio in adult HSV-2 genomes). The average dN/dS ratios for all proteins are listed in Table S2. Download FIG S4, TIF file, 0.6 MB. Copyright © 2019 Akhtar et al. 2019 Akhtar et al. This content is distributed under the terms of the Creative Commons Attribution 4.0 International license.

10.1128/mSphere.00590-18.8 TABLE S2 Number of nucleotide and amino acid (AA) differences observed in each set of neonatal or adult HSV-2 genomes. Download Table S2, XLSX file, 0.02 MB. Copyright © 2019 Akhtar et al. 2019 Akhtar et al. This content is distributed under the terms of the Creative Commons Attribution 4.0 International license.

### Minor variants expand the potential coding diversity of neonatal HSV-2 isolates.

**FIG 6:**
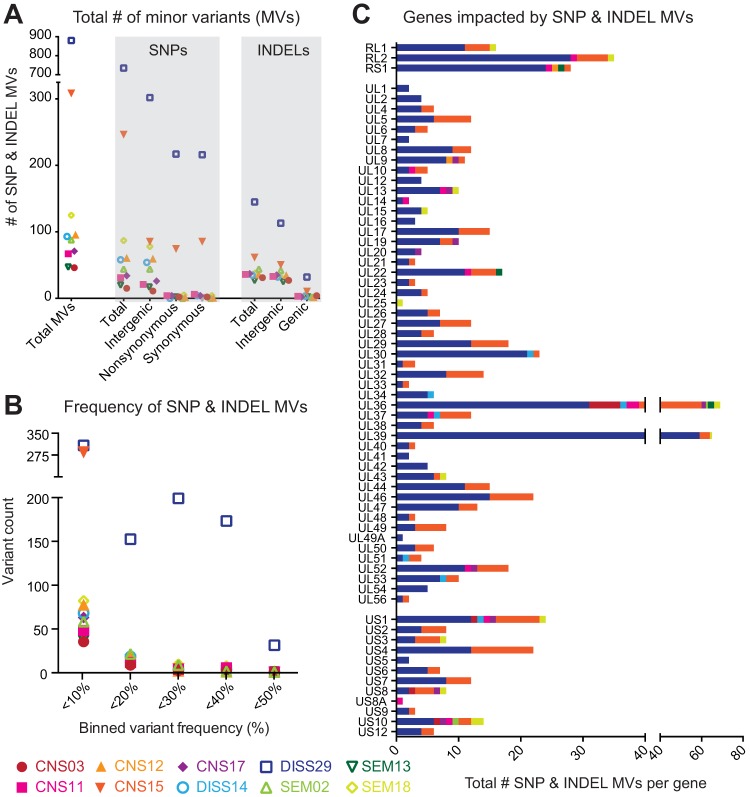
Minor variants expand the range of neonatal HSV-2 coding diversity. (A) Scatter plot indicates the total number of minor variants (MV; *y* axis) observed in each neonatal isolate. MV are rare alleles that exist within each viral population, at a frequency that is <50% but above a 2% limit of detection (see Materials and Methods for details). The total number of MV on the left is separated into single-nucleotide polymorphism (SNP) versus insertion/deletion (indel) variants on the right (*x* axis). The genomic location of each SNP or indel variant is also summarized as follows: intergenic versus inside genes (genic) for indels and intergenic versus nonsynonymous or synonymous SNPs inside genes. (B) The frequency, or penetrance, of each minor variant was examined for each isolate. Data (*x* axis) were binned in increments of 5% (e.g., 2% to <5% frequency, 5% to <10% frequency, and so on) and are plotted according to the number of MV observed at each frequency (*y* axis). SNP and indel variants were combined for this analysis. (C) Stacked histograms show the number of genic MV (*x* axis) located in each HSV-2 coding sequence (gene; *y* axis). SNP and indel variants were combined for this analysis. UL3, UL11, UL35, and UL55 lacked any minor variants and are not included in the histogram. See Table S3 for a full list of SNP and indel MV position and frequency data.

We next focused our attention on differences below the consensus level in each intrahost viral population. The amino acid variations described above exist in the consensus genomes of each isolate. Since viral replication creates a population of genomes, we next assessed whether minor allelic variants existed within the viral population of any neonatal HSV-2 isolate, thereby expanding the viral genetic diversity within each host. The significant depth of coverage from deep sequencing of each isolate allowed us to search for minor variants (MV) at every nucleotide position of each genome. We defined a minor variant as any nucleotide allele (single nucleotide polymorphism [SNP]) or insertion/deletion (indel) with a frequency level below 50% but above 2% (a conservative limit of detection; see Materials and Methods for additional criteria). We found minor variants in the viral genome population of all 10 neonatal HSV-2 isolates, albeit to different degrees in each isolate (Fig. 6A; see also Table S3). In total, there were 1,821 minor variants, distributed across all genomic regions (Fig. S5; see also Table S3). For both SNPs and indels, intergenic minor variants outnumbered those in genes (genic), likely reflecting the higher selective pressures against unfavorable mutations in coding regions. Neonatal isolate DISS29 had levels of minor variants that were 8-fold to 10-fold higher than those seen with the other neonatal isolates (Fig. 6A; see also Fig. S5), and those variants were often present at a higher frequency or with higher penetrance of the minor allele than was observed in other neonatal isolates (Fig. 6B; see also Fig. S5). We further examined the distribution of minor variants that occurred in genes and found that nearly every HSV-2 protein harbored minor variants in at least one neonatal isolate (Fig. 6C). Only UL3, UL11, UL35, and UL55 were completely devoid of minor variants. Three of these genes (UL3, UL11, and UL35) were also devoid of amino acid variations at the consensus level (Fig. 5). These data revealed the breadth of potential contributions of minor variants to neonatal HSV-2 biology, which could undergo selection over time or in specific niches.

10.1128/mSphere.00590-18.6 FIG S5 Genome-wide distribution and frequency of minor variants in neonatal HSV-2 isolates. For each neonatal HSV-2 isolate, the graph on the left plots spatial location in the genome (*x* axis) against the frequency at which each minor variant was observed. The plot on the right summarizes the number of minor variants (*y*-axis height) in binned increments of 1% (*x* axis). These data reveal the distinctly different distributions of minor variants in DISS29 and, to a lesser extent, CNS15 compared to other isolates. The color code matches that used in Fig. 6. See Table S3 for full list of SNP and indel MV position and frequency data. Download FIG S5, TIF file, 0.9 MB. Copyright © 2019 Akhtar et al. 2019 Akhtar et al. This content is distributed under the terms of the Creative Commons Attribution 4.0 International license.

10.1128/mSphere.00590-18.9 TABLE S3 Position and frequency of minor-variant SNPs and indels in neonatal HSV-2 genomes (two Excel tabs). Download Table S3, XLSX file, 0.2 MB. Copyright © 2019 Akhtar et al. 2019 Akhtar et al. This content is distributed under the terms of the Creative Commons Attribution 4.0 International license.

### Coding variations identified in neonatal HSV-2 isolates associated with CNS disease.

**FIG 7:**
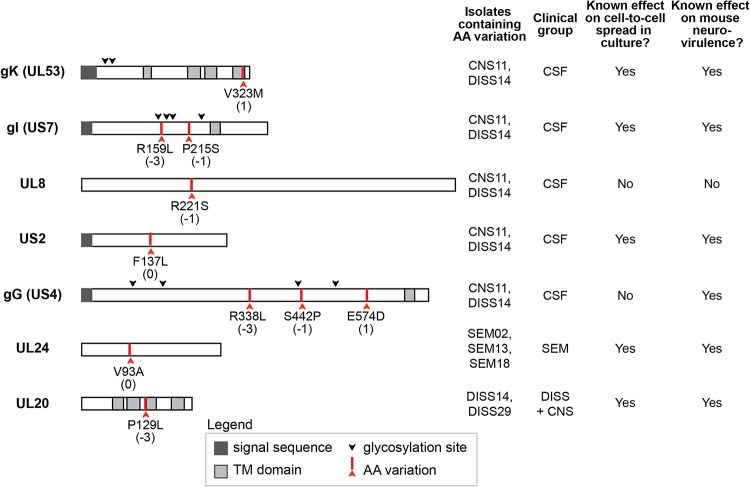
Several coding variations in neonatal HSV-2 isolates occur in proteins known to contribute to cell-to-cell spread and neurovirulence. The domain structure shown for each HSV protein is based on published literature for both HSV-1 and HSV-2. Red arrows and text labels indicate protein-coding variations discussed in the text, with the BLOSUM80 score for each amino acid substitution listed in parentheses beneath the text label. Detailed information and references for each protein on the domain structure and regarding cell-to-cell spread and neurovirulence can be found in Table S4.

To understand how viral genetic variants might relate to clinical disease, we assessed whether any of the consensus level coding variations identified in our group of 10 neonatal HSV samples segregated with clinical disease features. A number of amino acid variations were shared by CSF-derived isolates CNS11 and DISS14, which formed the largest plaques *in vitro*. These included amino acid variants at the level of the consensus genome in the HSV-2 genes encoding glycoprotein K (gK, UL53 gene: V323M), glycoprotein I (gI, US7 gene: R159L and P215S), UL8 (R221S), US2 (F137L), and glycoprotein G (gG, US4 gene: R338L, S442P, and E574D) (Fig. 7). The gI variants exist individually in other viral isolates obtained from neonates with CNS disease (Table S4). Isolates collected from infants experiencing disseminated disease with CNS involvement also shared a variant in the HSV-2 UL20 gene (P129L) (Fig. 7). One variant in the HSV-2 UL24 gene (V93A) was shared only by isolates from infants with SEM disease, with all isolates from infants with CNS disease containing a valine at this position (Fig. 7). The sample size of these comparisons was constrained by the overall limits of neonatal HSV-2 isolate availability and was too small to allow evaluation of statistical significance for any of these associations. While CNS11 and DISS14 shared several variants (listed in Fig. 7), these viral genomes were not similar at the consensus-genome level (Fig. 4). These isolates also had nonshared coding differences in the same genes that contained shared variants (e.g., gK, UL8, gG). This indicates a potential for convergent evolution at these loci. Many of the coding variations identified in this data set involve viral proteins that are known to modulate cell-to-cell spread (67–71) and/or contribute to neurovirulence in mouse models of CNS infection (69, 72–78) (Fig. 7; see also Table S4); however, their role in human disease has not yet been assessed.

10.1128/mSphere.00590-18.10 TABLE S4 Additional information and references for each viral protein shown in Fig. 7, including potential functions in cell-to-cell spread and/or neurovirulence. Download Table S4, PDF file, 0.2 MB. Copyright © 2019 Akhtar et al. 2019 Akhtar et al. This content is distributed under the terms of the Creative Commons Attribution 4.0 International license.

## DISCUSSION

Host factors have not been identified to explain the >50% of neonates experiencing invasive CNS or disseminated forms of HSV infection. There is growing evidence that most herpesviruses, including HSV-1 and HSV-2, contain significant genetic variation (27, 53, 56, 79–81). The potential contributions of viral genetic variation to clinical disease in neonates therefore warrant exploration. Here, we analyzed genetic and phenotypic diversity for HSV-2 isolated from 10 neonatal patients spanning two clinical studies (8, 9, 60). Although this group is small, the associations identified here provide the first insights into the potential impact of viral variability on clinical outcomes in neonatal HSV disease and serve as a starting point for further mechanistic investigation. We found that these 10 neonatal HSV-2 isolates exhibited diverse growth characteristics in culture, with larger-plaque-forming isolates observed more often in infants with CNS disease than in those with SEM disease. Furthermore, we established that enhanced viral spread through culture was the main contributor to this large-plaque formation. Using comprehensive comparative genomics, we further demonstrated that these neonatal HSV-2 isolates contained extensive genetic diversity both within and between hosts. These data revealed several specific viral genetic variations that were associated with cases of CNS disease in proteins known to contribute to cell-to-cell spread and/or neurovirulence in mouse models of CNS disease. Further studies are required to determine the impact of these variations on HSV-2 neurovirulence and progression to CNS disease.

Genomic comparison of these neonatal isolates revealed a wide range of genetic diversity. At the consensus-genome level, which reflects the most common allele in each viral population, we found that the coding differences between strains were as numerous as those between previously described sets of adult HSV-2 isolates (Fig. 5) (79–81). Furthermore, the specific genetic variations associated with neonatal CNS disease in our study can also be found in genital HSV-2 isolates which are not associated with CNS disease in adults. This suggests that the dramatic differences in clinical manifestations seen following HSV-2 infection in neonates, with significantly higher rates of invasive CNS infection, are not due to unique neonatal HSV-2 strains. However, we did detect several outliers in the comparison between the rates of nonsynonymous to synonymous nucleotide differences (the dN/dS ratio) for several genes in neonatal HSV-2 isolates versus those previously sequenced from adult patients (see Fig. S4). The genes encoding putative virulence factor US8A (82, 83) and capsid triplex protein VP19C (UL38) (84, 85) have a markedly higher dN/dS ratio in neonates, while that of glycoprotein C (gC; UL44) is notably lower in neonates than in adults (Fig. S4). These differences in dN/dS ratios may reflect the distinct host environment of these isolates. For example, viruses isolated from adult patients, often with recurrent genital infection, may have increased diversity of surface proteins such as gC due to selection for viral immune evasion (86, 87). The lower variability in gC (UL44) in neonates could result from their immunologically naive state and/or from the shorter duration of neonatal infection prior to virus isolation (Table 1). The observation of certain genes having higher dN/dS ratios in neonatal isolates than in adults could have resulted either from a loosening of selective pressures (i.e., drift) or from driving forces unique to the neonatal environment that remain to be understood. Comparisons of additional isolates and ongoing characterization of understudied proteins such as US8A (83) will help to distinguish these possibilities.

At the level of specific genetic variations in individual proteins, we detected a few fully penetrant patterns that distinguish one clinical group from another. All of the fully penetrant, group-specific variations that we detected in the two CSF-derived strains, the two disseminated strains, and three SEM strains available for study are highlighted in Fig. 7. However, other loci of potential interest exist if we consider those genetic variants found in a majority, but not all, of the neuroinvasive (CNS and DISS) strains—e.g., variations in envelope glycoprotein gH (UL22), viral serine/threonine kinase US3, viral thymidine kinase (UL23), major capsid protein UL19, and DNA polymerase processivity factor UL42. Characterization of additional neonatal isolates will no doubt improve the clarity of these comparisons and help to distinguish consistent patterns from those detected by chance due to the clinical limitations of the infant sample pool. Regardless of the specific viral genetic candidates under consideration, we hypothesize that viral variations impacting neonatal outcomes could act either by conferring enhanced neurovirulence or by limiting the rate of viral spread or degree of neuroinvasion. For instance, it is tempting to speculate that variations in glycoprotein I (US7) might be associated with enhanced viral spread, due to their observation in several large-plaque-associated viruses in this study and to prior data demonstrating the involvement of gI in both immune evasion and neuronal spread (see Table S4) (68, 88, 89). Conversely, the UL24 V93A variant, which was observed only in SEM-derived isolates and is relatively rare among adult HSV-2 isolates as well, could be a potential example of a spread-limiting variation. UL24 function is required to disperse nucleolin during lytic HSV-1 infection (90, 91), and mutation of UL24 has been associated with a loss of neuroinvasion in animal models (75–77). Further research will be needed to build stronger genetic associations with additional neonatal isolates and to expand upon prior studies by testing these specific genetic variations in animal models. The application of animal models of neonatal infection will also enable the exploration of how nongenetic environmental factors such as dose and timing of viral exposure contribute to the severity and progression of disease and how viral genetic variations and environmental factors intersect with the host genetic background.

At the level of minor variants (MV), which represent rare alleles that exist within each intrahost viral population, we found that the DISS29 genome harbored 8-fold to 10-fold more and the CNS15 genome harbored 3-fold to 4-fold more MV than other neonatal virus genomes (Fig. 6; see also Fig. S4). This could be indicative of a mixed viral population (e.g., a multistrain infection), decreased polymerase fidelity, or the presence or absence of host selective pressure (27, 28). Diversity in viral populations has also been observed in congenital HCMV infection (30–35). However, this is the first time that evidence has been found for this level of viral population diversity with HSV-2. These minor genotypes may be selected or genetically isolated in particular niches (e.g., CSF), as observed in a comparison of VZV skin vesicles (26), or by antiviral drug selection, as was recently demonstrated in two adults with genital HSV-2 infection (40). All of the isolates sequenced in this study were collected at the initial time of diagnosis (from neonates who were ≤19 days old), prior to acyclovir treatment and prior to the development of an HSV-specific immune response. It would therefore be compelling to examine the viral genome population from serial patient isolates over time to identify shifts in the frequency of MV due to antiviral or immune selection. Isolates from different body sites of the same patient could likewise be compared to determine whether particular genotypes are enriched in different body niches.

This comparison of viral genotype to clinical phenotype revealed associations between neonatal CNS disease and several viral protein variants that may impact neurovirulence through modulation of cell-to-cell spread. Although the sample set in this proof-of-concept study was small, we observed potential patterns that warrant exploration in a larger data set. It must be acknowledged that there is limited availability of samples from neonatal infection due to both the rarity of these infections and the fragility and small physical size of the infected infants. These natural circumstances lead to minimal sample collection from infected neonates. The finding that the CNS-associated isolates in our study, particularly those derived directly from the CSF, often exhibited enhanced spread between cells in culture suggests that one or more of these variants could be functionally significant. Coding differences in viral proteins not known to contribute to neurovirulence were also found to be associated with neonatal CNS disease and represent potential novel contributions to invasive infection. These promising results warrant exploration in a larger study, ideally one analyzing isolates from multiple time points and/or body sites from each infected infant. This would enable a better understanding of how overall viral genetic diversity contributes to neuroinvasion.

## MATERIALS AND METHODS

### Viruses.

Viruses were collected from neonates enrolled in clinical studies (8, 9, 60) by the Collaborative Antiviral Study Group (CASG) at the University of Alabama at Birmingham (UAB). Samples were collected from either the cerebrospinal fluid (CSF) or skin. Enrollment in original studies was evenly split between males and females and included both black and white patients. The clinical morbidity score was determined at 12 months of life as previously defined (12, 92). Initial collection of samples, use of samples in this study, and use of deidentified clinical information were approved by the UAB Institutional Review Board. See Text S1 in the supplemental material for additional details of this and subsequent methods.

10.1128/mSphere.00590-18.1 TEXT S1 Text file with additional methodological details. Download Text S1, PDF file, 0.2 MB. Copyright © 2019 Akhtar et al. 2019 Akhtar et al. This content is distributed under the terms of the Creative Commons Attribution 4.0 International license.

### Cell culture.

Human lung fibroblast MRC-5 cells (ATCC, CCL-171), African green monkey kidney Vero cells (ATCC, CCL-81), and human epithelial bone osteosarcoma U2OS cells (ATCC, HTB-96) were cultured under standard conditions. Cell lines were authenticated by ATCC prior to purchase and were confirmed to be mycoplasma free throughout the experiments by periodic testing (LookOut Mycoplasma; Sigma).

### Virus culture.

Viruses were cultured at the time of diagnosis and snap-frozen after 1 passage. Each viral isolate was then passaged 3 times on MRC-5 cells at an MOI of 0.01, with harvest at the time of complete cytopathic effect (between 50 to 70 h). Titers of viral stocks were determined on either Vero cells (100 h) or U2OS cells (48 h) under a methylcellulose overlay. Plaque size and morphology did not change for any viral isolates over the course of virus stock expansion.

### Plaque measurements.

Plaques were stained with 0.5% methylene blue. Serial 4× bright-field images were collected on an EVOS FL Auto Imaging system and stitched by EVOS software (University of Pennsylvania [UPenn] Cell and Developmental Biology Microscopy Core). Plaque area was measured using ImageJ software.

### Genome copy number estimation by quantitative PCR for UL27.

DNA was extracted and quantitative PCR was performed with primers specific to the viral glycoprotein B gene (gB; UL27) (43). Absolute quantification was calculated based on a standard curve of HSV-1 strain F nucleocapsid DNA (59).

### Viral entry assay.

Monolayers of Vero cells were cooled to 4°C for 30 min prior to infection with 100 PFU of each viral isolate. After 1 h of viral incubation at 4°C, unbound virus was removed by washing and cells were moved to 37°C. At 0, 10, 20, 30, 45, or 60 min, a low-pH citrate buffer was applied to inactivate extracellular virus. Under each condition, parallel infections were performed without the addition of citrate solution (control). Cell monolayers were washed and allowed to form plaques under methylcellulose for 100 h. Viral entry was quantified as the fraction of plaques formed following citrate buffer application, where 100% is the number of plaques formed on a monolayer not treated with citrate buffer (control).

### Single-step and multistep growth curves.

Growth curves of HSV infection were performed in Vero cells. At 2 hpi, 0.1% human serum was added to reduce cell-free spread of virus. Single-step growth curves were performed at MOI = 5, and multistep growth curves at MOI = 0.001, as defined by titering viral stocks on U2OS cells. Every 24 h, the supernatant was removed and media containing 0.1% human serum reapplied.

### Immunocytochemistry.

Immunocytochemistry analysis was performed as previously described (93). Infection was detected with rabbit anti-HSV primary antibodies (Agilent Dako; B0114) and fluorophore-conjugated anti-rabbit secondary antibodies (Invitrogen; A-11008). Cell nuclei were counterstained with DAPI (4′,6-diamidino-2-phenylindole). Images (5×) were collected with a Leica DM6000 wide-field microscope equipped with a Photometrics HQ2 high-resolution monochrome charge-coupled-device (CCD) camera and processed with LAS AF software (UPenn Cell and Developmental Microscopy Core). Images (10×) were collected on an EVOS FL Auto Imaging system and stitched using EVOS software (UPenn Cell and Developmental Microscopy Core). Exposure and gain were optimized within each experiment for one virus at the 72-h time point and applied identically to each image within that experiment. Subsequent image processing (ImageJ) was applied equally to all images in a given experiment.

### Immunoblotting.

Immunoblotting was performed as previously described (93). Equal amounts of whole-cell lysate were separated by SDS-PAGE. Membranes were immunoblotted with antibodies raised against total HSV (Agilent Dako, B0114); glycoprotein C (gC), gD, gE, gH, and VP5 (all gifts from Gary Cohen); ICP8 (gift from David Knipe); and GAPDH (glyceraldehyde-3-phosphate dehydrogenase) (GeneTex; GTX100118).

### Viral DNA isolation and Illumina sequencing.

Viral nucleocapsid DNA for genome sequencing was prepared by infecting MRC-5 cells at an MOI of ≥5 as previously described (94, 95). Viral nucleocapsid gDNA was sheared using a Covaris M220 sonicator/disruptor under the following conditions: duration, 60 s; peak power, 50; duty cycle, 10%; temperature, 4°C. Barcoded sequencing libraries were prepared using the Illumina TruSeq low-throughput protocol according to manufacturer’s specifications and as previously described (54, 57). The quality of sequencing libraries was evaluated by Qubit (Invitrogen, CA), Bioanalyzer (Agilent), and qPCR (KAPA Biosystems). Paired-end sequencing (2 × 300-bp length) was performed on an Illumina MiSeq, according to manufacturer’s recommendations (17 pM input).

### *De novo* genome assembly.

A consensus genome was assembled for each viral isolate using a previously described Viral Genome Assembly (VirGA) bioinformatics workflow (54) (Table 2). Annotation of new genome sequences was guided by the use of the HSV-2 reference genome (strain HG52; GenBank accession no. NC_001798) based on sequence homology (96).

### Comparative genomics and phylogenetic analysis.

The 10 neonatal HSV-2 genomes were aligned with all annotated HSV-2 genomes available in GenBank (see Table S1 for the full list of 58 genomes, all of which were derived from adults) using MAFFT (97). The genome-wide alignment used a trimmed-genome format (lacking the terminal repeats) to avoid giving undue weight to the duplicated sequences. The MAFFT alignment was used to generate a NeighborNet phylogenetic network in SplitsTree with uncorrected *P* distances (49, 98, 99), as well as a neighbor-joining tree (Jukes-Cantor; 1,000 bootstraps) in MEGA Omega for Windows (100). A diverse subset of 10 adult HSV-2 isolates was selected for protein-level comparisons with the 10 neonatal isolates (indicated in Table S1 with an asterisk). ClustalW2 was used to construct pairwise nucleotide alignments between whole genomes and to construct pairwise amino acid alignments for each gene and protein (101). Pan-HSV-2 comparisons excluded the following three viral proteins for which sequences have not been fully determined in most published strains, likely due to the high levels of G+C content and numerous tandem repeats in these regions: ICP34.5 (RL1—annotated/complete in only 9 published genomes in Table S1), ICP0 (RL2—annotated/complete in only 8 genomes in Table S1), and ICP4 (RS1—annotated/complete in only 6 genomes in Table S1) (79–81). Custom Python scripts were used on these alignments to identify nucleotide and amino acid differences between samples.

### Minor variant detection and quantification.

Minor variants (MV) were detected using VarScan v2.2.11 (mpileup2snp and mpileup2indel commands) (102) and the following parameters to differentiate true MV from technical artifacts (26): minimum allele frequency of ≥0.02 (2%); base call quality of ≥20; read depth of ≥100; ≥5 independent reads supporting minor alleles (see Table S3). MV with directional strand bias levels of ≥90% were excluded. The genomic location and potential impact of each MV were assessed using SnpEff and SnpSift (103, 104). We use the conservative cutoff value of 2% for detection of minor variants both to avoid false-positive signals and to provide data that are comparable to recent examples in the literature on genomic analysis of herpesviruses in clinical samples (28, 105, 106).

### Data availability.

Newly deposited sequences for HSV-2 isolates can be found in GenBank under accession no. MK105995 to MK106004.
