# Supplementary figures and images for: Genotypic and Phenotypic Diversity of Herpes Simplex Virus 2 within the Infected Neonatal Population
Source: mSphere. 2019 Feb 27;4(1):e00590-18. doi: 10.1128/mSphere.00590-18 (PMC6393728; doi:10.1128/mSphere.00590-18)

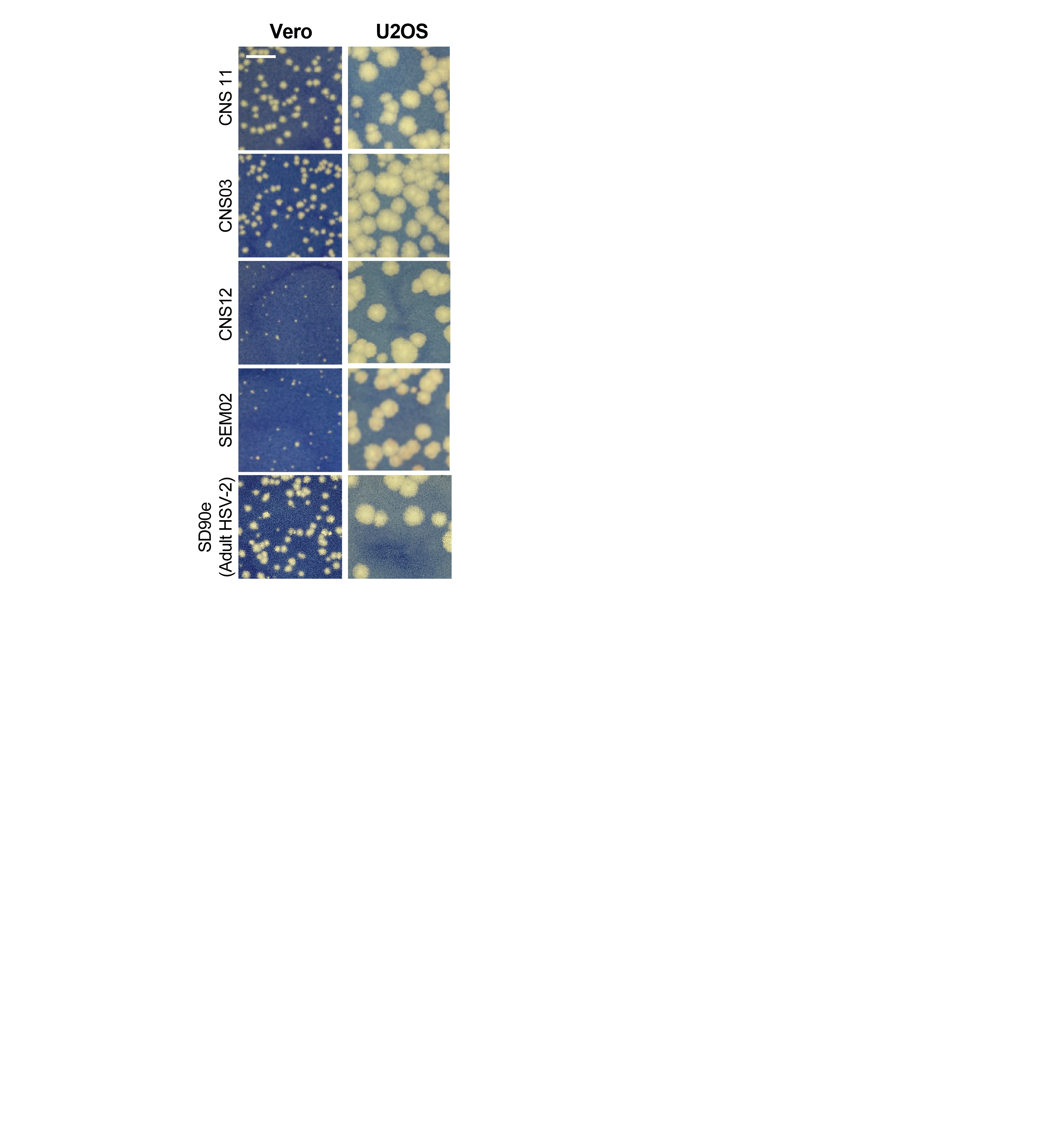

Supplement: FIG S1 [file mSphere.00590-18-sf001.tif]

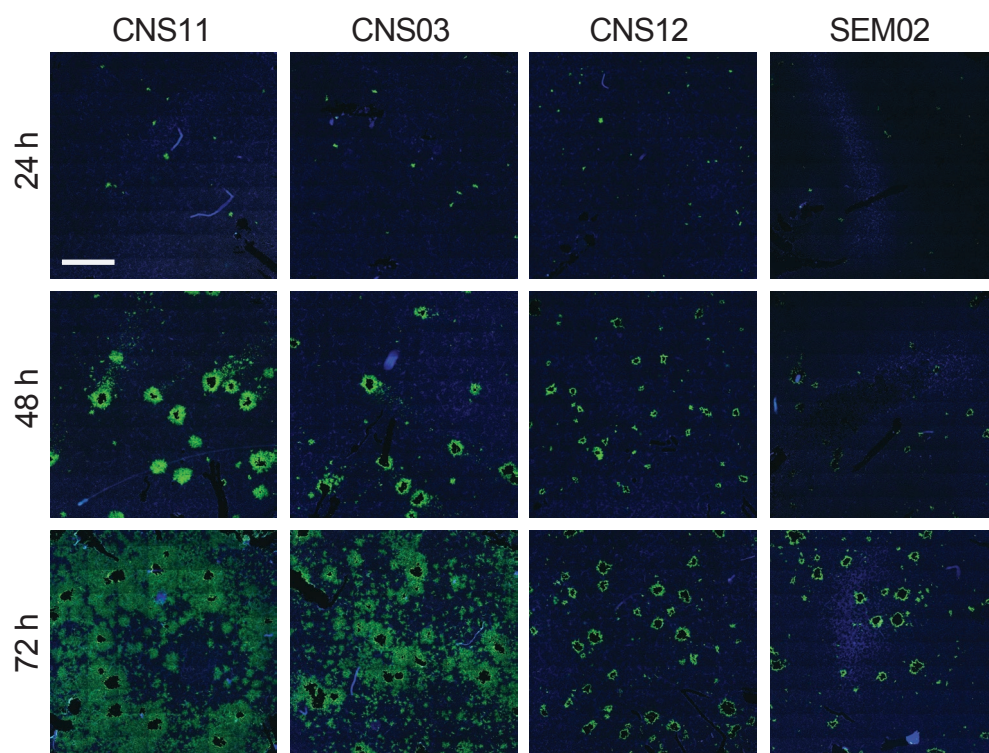

Supplement: FIG S2 [file mSphere.00590-18-sf002.pdf]

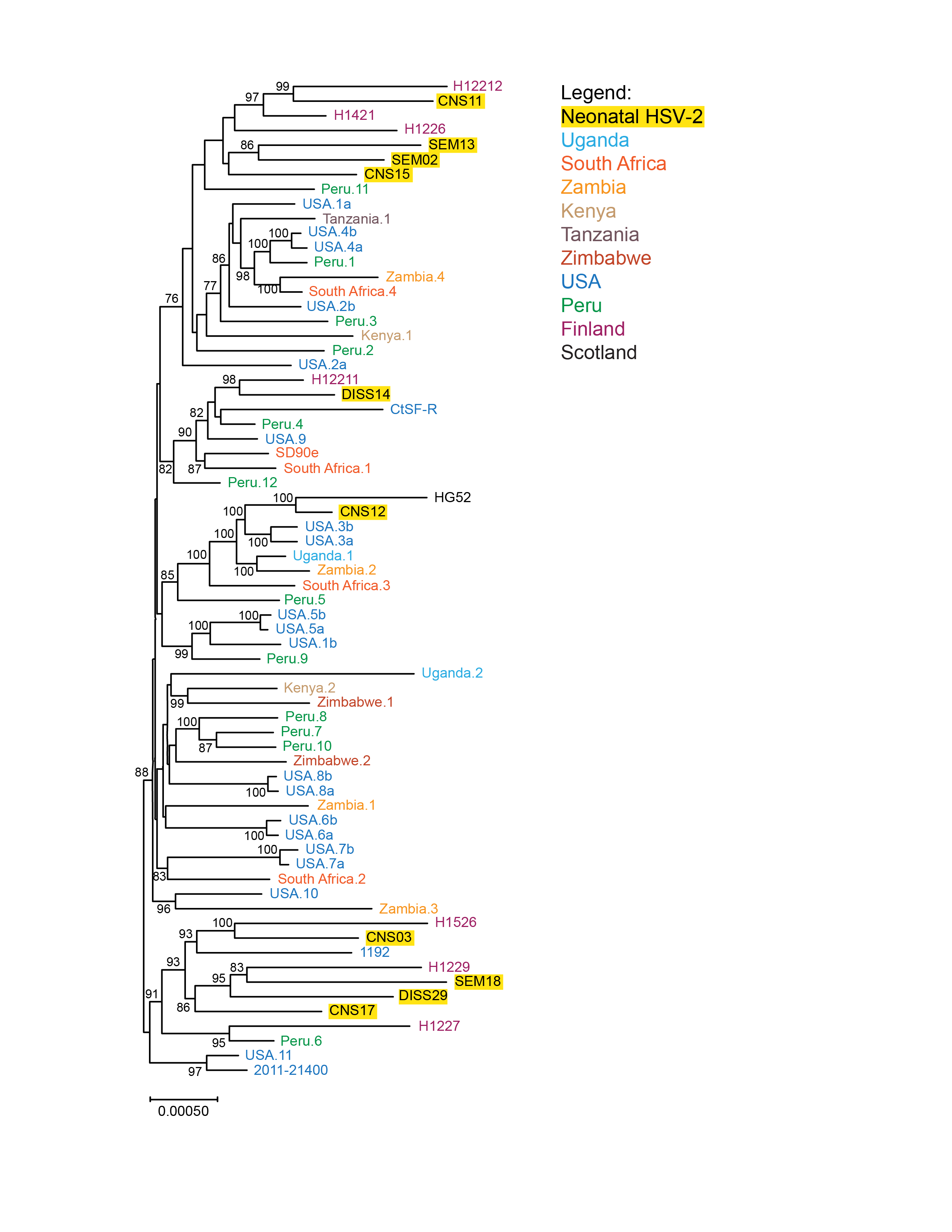

Supplement: FIG S3 [file mSphere.00590-18-sf003.tif]

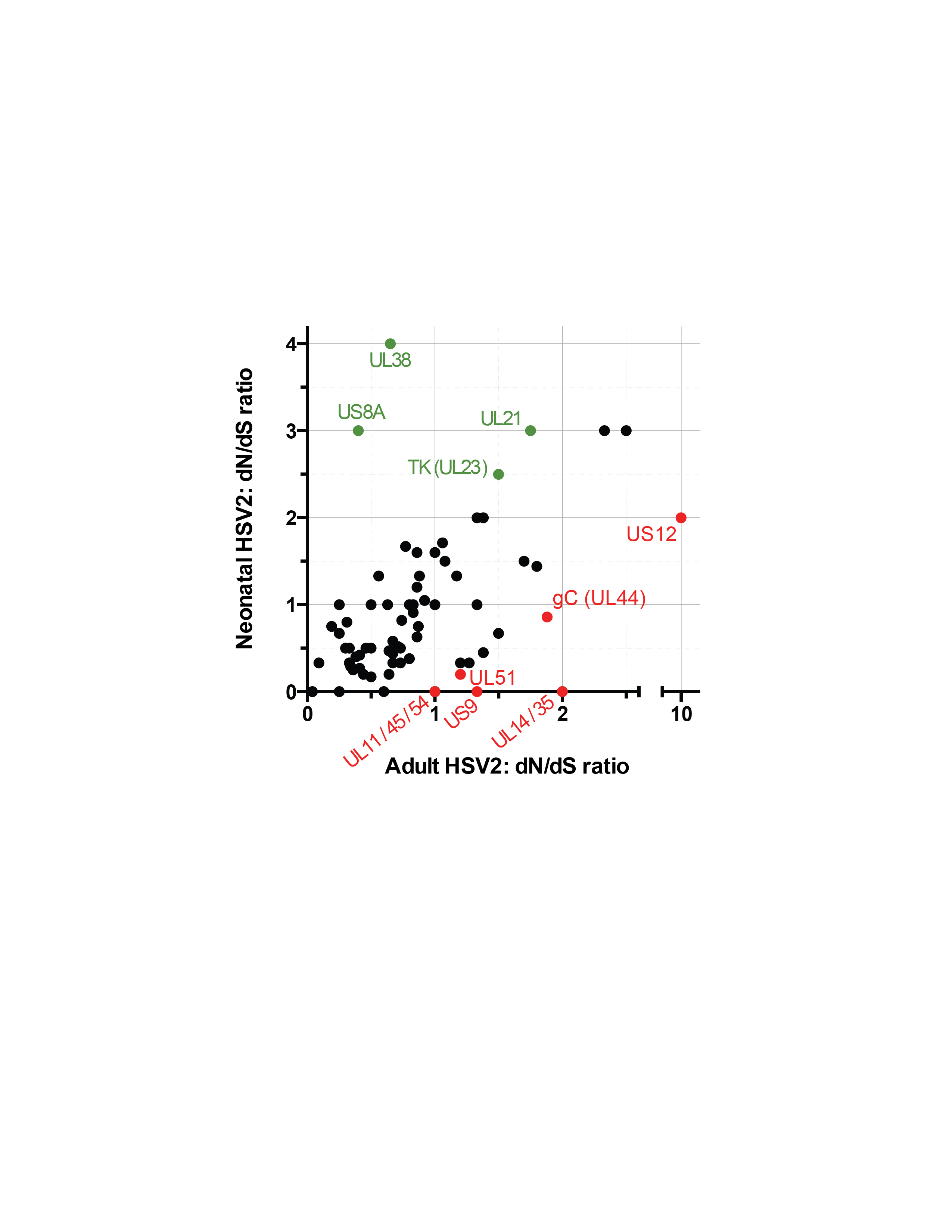

Supplement: FIG S4 [file mSphere.00590-18-sf004.tif]

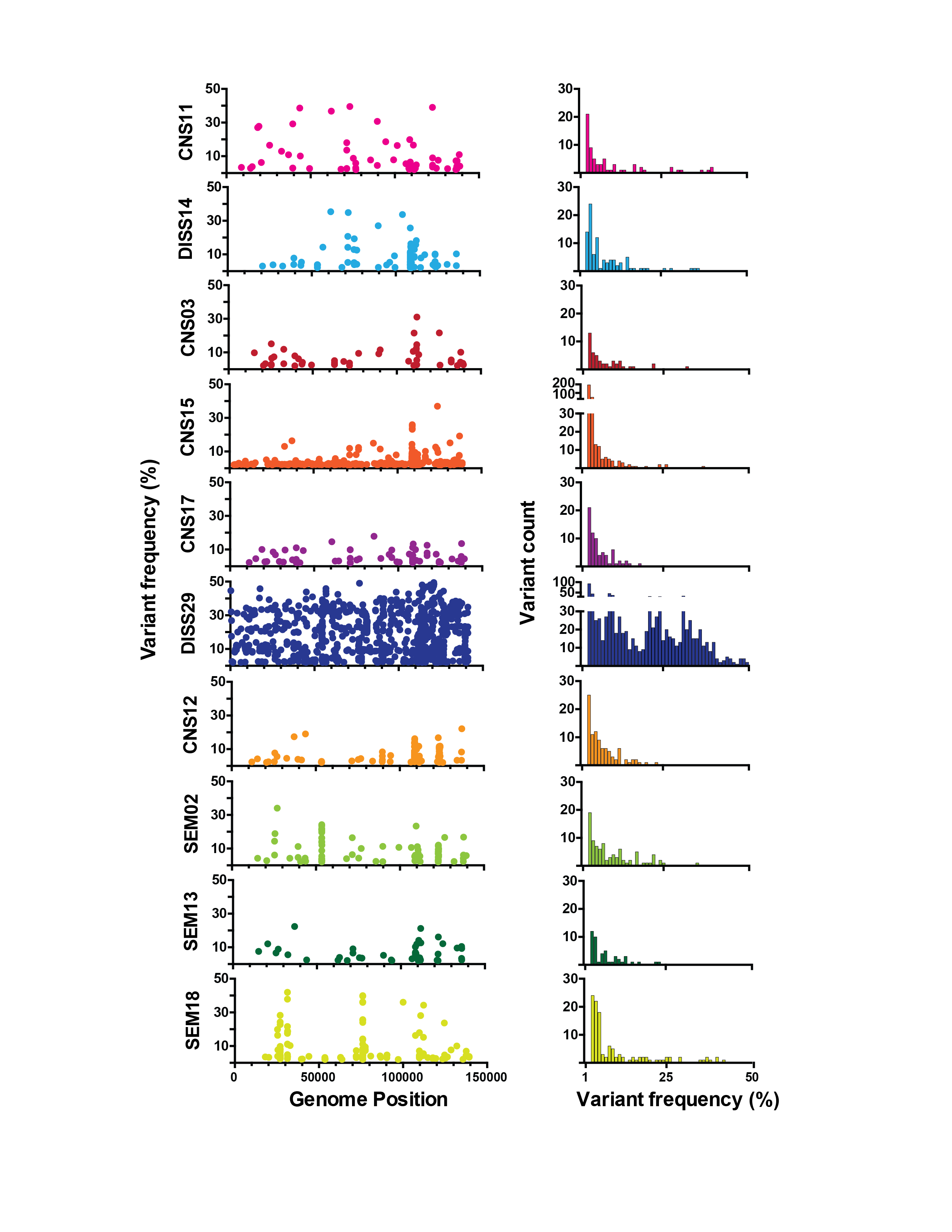

Supplement: FIG S5 [file mSphere.00590-18-sf005.tif]
