# Supplementary material for: Genotypic and Phenotypic Diversity of Herpes Simplex Virus 2 within the Infected Neonatal Population
Source: mSphere. 2019 Feb 27;4(1):e00590-18. doi: 10.1128/mSphere.00590-18 (PMC6393728; doi:10.1128/mSphere.00590-18)
Supplement: TABLE S1 [file mSphere.00590-18-st001.pdf]

Supplemental Table S1. Adult HSV-2 genomes (58 total) used for comparative genomic analyses.

Virus Isolate	Country (with location, if available)	GenBank Accession #	References
H1227*	Finland	KY922721	In process
H1229*	Finland	KY922722	In process
H12212*	Finland	KY922726	In process
Uganda.2*	Uganda	KX574906	(1)
Peru.8*	Peru	KX574874	(1)
Zambia.4*	Zambia	KX574883	(1)
HG52*	Scotland	NC_001798	(2)
CtSF-R*	USA	KP334093	(3)
1192*	Wisconsin, USA	KP334095	(3)
SD90e*	Carletonville, South Africa	KF781518	(4, 5)
Peru.1	Peru	KX574860	(1, 6)
Peru.12	Peru	KX574861	(1, 6)
Zambia.3	Zambia	KX574862	(1, 6)
Zimbabwe.2	Zimbabwe	KX574863	(1, 6)
Zambia.2	Zambia	KX574864	(1, 6)
Peru.9	Peru	KX574865	(1, 6)
Peru.11	Peru	KX574866	(1, 6)
Peru.10	Peru	KX574867	(1, 6)
USA.8a	USA	KX574868	(1, 6)
USA.8b	USA	KX574869	(1, 6)
USA.1b	USA	KX574870	(1, 6)
Zimbabwe.1	Zimbabwe	KX574871	(1, 6)
Peru.7	Peru	KX574872	(1, 6)
Peru.2	Peru	KX574873	(1, 6)
Peru.6	Peru	KX574875	(1, 6)
Peru.4	Peru	KX574876	(1, 6)
Peru.3	Peru	KX574877	(1, 6)
Peru.5	Peru	KX574878	(1, 6)
South Africa.2	South Africa	KX574879	(1, 6)
South Africa.3	South Africa	KX574880	(1, 6)
South Africa.4	South Africa	KX574881	(1, 6)
USA.3a	USA	KX574882	(1, 6)
USA.3b	USA	KX574884	(1, 6)
USA.2b	USA	KX574885	(1, 6)
USA.10	USA	KX574886	(1, 6)
USA.4a	USA	KX574887	(1, 6)
USA.7a	USA	KX574888	(1, 6)

Virus Isolate	Country (with location, if available)	GenBank Accession #	References
USA.6a	USA	KX574889	(1, 6)
USA.6b	USA	KX574890	(1, 6)
USA.5a	USA	KX574891	(1, 6)
South Africa.1	South Africa	KX574892	(1, 6)
Kenya.2	Kenya	KX574893	(1, 6)
Kenya.1	Kenya	KX574894	(1, 6)
USA.7b	USA	KX574895	(1, 6)
USA.5b	USA	KX574896	(1, 6)
USA.11	USA	KX574897	(1, 6)
USA.4b	USA	KX574898	(1, 6)
Uganda.1	Uganda	KX574899	(1, 6)
USA.9	USA	KX574901	(1, 6)
Tanzania.1	Tanzania	KX574902	(1, 6)
USA.1a	USA	KX574903	(1, 6)
USA.2a	USA	KX574904	(1, 6)
Zambia.1	Zambia	KX574905	(1, 6)
2011-21400	USA	KX574908	(1, 6)
H12211	Finland	KY922725	In process
H1226	Finland	KY922720	In process
H1421	Finland	KY922723	In process
H1526	Finland	KY922724	In process

*Indicates 10 adult HSV-2 strains used for AA comparisons in Figure 5.

References for Supplemental Table S1

1. Johnston C, Magaret A, Roychoudhury P, Greninger AL, Cheng A, Diem K, Fitzgibbon MP, Huang M, Selke S, Lingappa JR, Celum C, Jerome KR, Wald A, Koelle DM. 2017. Highly conserved intragenic HSV-2 sequences: Results from next-generation sequencing of HSV-2 U L and U S regions from genital swabs collected from 3 continents. Virology 510:90–98.

2. Davison AJ. 2012. Evolution of sexually transmitted and sexually transmissible human herpesviruses. Ann N Y Acad Sci 1230:E37–E49.

3. Kolb AW, Larsen IV, Cuellar JA, Brandt CR. 2015. Genomic, Phylogenetic, and Recombinational Characterization of Herpes Simplex Virus 2 Strains. J Virol 89:6427–6434.

4. Colgrove R, Diaz F, Newman R, Saif S, Shea T, Young S, Henn M, Knipe DM. 2014. Genomic sequences of a low passage herpes simplex virus 2 clinical isolate and its plaque-purified derivative strain. Virology 450–451:140–145.

5. Lai W, Chen CY, Morse SA, Htun Y, Fehler HG, Liu H, Ballard RC. 2003. Increasing relative prevalence of HSV-2 infection among men with genital ulcers from a mining community in South Africa. Sex Transm Infect 79:202–207.

6. Koelle DM, Norberg P, Fitzgibbon MP, Russell RM, Greninger AL, Huang M-L, Stensland L, Jing L, Magaret AS, Diem K, Selke S, Xie H, Celum C, Lingappa JR, Jerome KR, Wald A, Johnston C. 2017. Worldwide circulation of HSV-2 × HSV-1 recombinant strains. Sci Rep 7:44084.
