# Supplementary material for: Genotypic and Phenotypic Diversity of Herpes Simplex Virus 2 within the Infected Neonatal Population
Source: mSphere. 2019 Feb 27;4(1):e00590-18. doi: 10.1128/mSphere.00590-18 (PMC6393728; doi:10.1128/mSphere.00590-18)
Supplement: TABLE S4 [file mSphere.00590-18-st004.pdf]

Supplemental Table S4. Viral genetic variations potentially associated with clinical groups.

HSV-2 Gene: Variation	Clinical Group (Isolates w/ Variation)	Predicted effect^	Protein Function	Neurovirulence
**gK (UL53)**: V323M	All CSF Isolates (CNS11, DISS14)	Loss of one TM domain	Glycoprotein at virion and cell surface; interacts with gB and UL20 (1); important for cytoplasmic envelopment, viral egress, and virus-induced cell fusion	gK null virus exhibits poor neuronal spread in culture including decreased retrograde and anterograde transport (2); reduced spread within eye and to nervous system, as well as increased survival, following ocular challenge in mice (3)
**gI (US7)**: R159L + P215S	All CSF Isolates (Both: CNS11,DISS14 R159L only: CNS03, SEM13,CNS15*,DISS29* P215S only: CNS15*,DISS29)	R159L: adds one TM domain; P215S: no change	Glycoprotein at virion and cell surface; forms heterodimer with gE to form viral Fc receptor (4); required for cell-to-cell spread of virus in epithelial and neuronal cells (5, 6);	gI null virus exhibits poor neuronal spread in culture; reduced spread in retina and to retinorecipient areas of the brain following ocular challenge in mice (6)
**UL8**: R221S	All CSF Isolates (CNS11, DISS14)	No predicted change	Putative primase subunit of helicase primase complex; required for unwinding of viral DNA	None identified
**US2**: F137L	All CSF Isolates (CNS11, DISS14)	No predicted change	Tegument protein; binds and phosphorylates TAK1 to positively regulate NFkB signaling (7)	Virus containing mutated US2 forms small plaques in culture (8); Mutant virus containing a deletion of both US2 and PK result in decreased death following intracerebral injection in mice (9)
**gG (US4)**: R338L,S442P, E574D	All CSF Isolates (CNS11, DISS14)	No predicted change	Binds and enhances function of chemokines (10); enhances neurotrophin-dependent axonal growth in culture (11)	Virus containing mutated gG results in decreased death following intracerebral injection in mice (8)
**UL24**: V93A	No CNS Isolates; only found in SEM isolates (SEM02, SEM13, SEM18)	No predicted change	Membrane-associated nuclear protein; associated with dispersal of nucleolin during infection (12, 13)	UL24 null virus forms small plaques in culture (14); decreased viral load in eye and nervous system following ocular challenge in mice (15–17)
**UL20**: P129L	All DISS Isolates w/ CNS Involved (DISS14, DISS29)	Change in position of three TM domains	Membrane-associated protein interacts with gK and gB (1); required for gK glycosylation, cell surface expression (18), and virus-induced cell fusion (19); important for viral egress (20)	UL20 null virus forms small plaques in culture (19); RNA inhibition of UL20 results in decreased rates of encephalitis following HSV footpad infection in mice (21)

* present as minor variant ^ Prediction by SMART Analysis

References for Supplemental Table S4

1. Foster TP, Chouljenko VN, Kousoulas KG. 2008. Functional and physical interactions of the herpes simplex virus type 1 UL20 membrane protein with glycoprotein K. J Virol 82:6310–6323.

2. David AT, Saied A, Charles A, Subramanian R, Chouljenko VN, Kousoulas KG. 2012. A Herpes Simplex Virus 1 (McKrae) Mutant Lacking the Glycoprotein K Gene Is Unable To Infect via Neuronal Axons and Egress from Neuronal Cell Bodies. mBio 3.

3. David AT, Baghian A, Foster TP, Chouljenko VN, Kousoulas KG. 2008. The Herpes Simplex Virus Type 1 (HSV-1) Glycoprotein K(gK) is Essential for Viral Corneal Spread and Neuroinvasiveness. Curr Eye Res 33:455–467.

4. Johnson DC, Frame MC, Ligas MW, Cross AM, Stow ND. 1988. Herpes simplex virus immunoglobulin G Fc receptor activity depends on a complex of two viral glycoproteins, gE and gI. J Virol 62:1347–1354.

5. Dingwell KS, Brunetti CR, Hendricks RL, Tang Q, Tang M, Rainbow AJ, Johnson DC. 1994. Herpes simplex virus glycoproteins E and I facilitate cell-to-cell spread in vivo and across junctions of cultured cells. J Virol 68:834–845.

6. Dingwell KS, Doering LC, Johnson DC. 1995. Glycoproteins E and I facilitate neuron-to-neuron spread of herpes simplex virus. J Virol 69:7087–7098.

7. Lu X, Huang C, Zhang Y, Lin Y, Wang X, Li Q, Liu S, Tang J, Zhou L. 2017. The Us2 Gene Product of Herpes Simplex Virus 2 modulates NF-κB activation by targeting TAK1. Sci Rep 7:8396.

8. Weber PC, Levine M, Glorioso JC. 1987. Rapid identification of nonessential genes of herpes simplex virus type 1 by Tn5 mutagenesis. Science 236:576–579.

9. Meignier B, Longnecker R, Mavromara-Nazos P, Sears AE, Roizman B. 1988. Virulence of and establishment of latency by genetically engineered deletion mutants of herpes simplex virus 1. Virology 162:251–254.

10. Viejo-Borbolla A, Martinez-Martín N, Nel HJ, Rueda P, Martín R, Blanco S, Arenzana-Seisdedos F, Thelen M, Fallon PG, Alcamí A. 2012. Enhancement of chemokine function as an immunomodulatory strategy employed by human herpesviruses. PLoS Pathog 8:e1002497.

11. Cabrera JR, Viejo-Borbolla A, Martinez-Martín N, Blanco S, Wandosell F, Alcamí A. 2015. Secreted herpes simplex virus-2 glycoprotein G modifies NGF-TrkA signaling to attract free nerve endings to the site of infection. PLoS Pathog 11:e1004571.

12. Lymberopoulos MH, Pearson A. 2007. Involvement of UL24 in herpes-simplex-virus-1-induced dispersal of nucleolin. Virology 363:397–409.

13. Bertrand L, Leiva-Torres GA, Hyjazie H, Pearson A. 2010. Conserved Residues in the UL24 Protein of Herpes Simplex Virus 1 Are Important for Dispersal of the Nucleolar Protein Nucleolin. Author Correction. J Virol 84:10436.

14. Jacobson JG, Martin SL, Coen DM. 1989. A conserved open reading frame that overlaps the herpes simplex virus thymidine kinase gene is important for viral growth in cell culture. J Virol 63:1839–1843.

15. Rochette P-A, Bourget A, Sanabria-Solano C, Lahmidi S, Lavallée GO, Pearson A. 2015. Mutation of UL24 impedes the dissemination of acute herpes simplex virus 1 infection from the cornea to neurons of trigeminal ganglia. J Gen Virol 96:2794–2805.

16. Leiva-Torres GA, Rochette P-A, Pearson A. 2010. Differential importance of highly conserved residues in UL24 for herpes simplex virus 1 replication in vivo and reactivation. J Gen Virol 91:1109–1116.

17. Jacobson JG, Chen SH, Cook WJ, Kramer MF, Coen DM. 1998. Importance of the herpes simplex virus UL24 gene for productive ganglionic infection in mice. Virology 242:161–169.

18. Foster TP, Alvarez X, Kousoulas KG. 2003. Plasma membrane topology of syncytial domains of herpes simplex virus type 1 glycoprotein K (gK): the UL20 protein enables cell surface localization of gK but not gK-mediated cell-to-cell fusion. J Virol 77:499–510.

19. Foster TP, Melancon JM, Baines JD, Kousoulas KG. 2004. The herpes simplex virus type 1 UL20 protein modulates membrane fusion events during cytoplasmic virion morphogenesis and virus-induced cell fusion. J Virol 78:5347–5357.

20. Foster TP, Melancon JM, Olivier TL, Kousoulas KG. 2004. Herpes simplex virus type 1 glycoprotein K and the UL20 protein are interdependent for intracellular trafficking and trans-Golgi network localization. J Virol 78:13262–13277.

21. Liu J, Lewin AS, Tuli SS, Ghivizzani SC, Schultz GS, Bloom DC. 2008. Reduction in severity of a herpes simplex virus type 1 murine infection by treatment with a ribozyme targeting the UL20 gene RNA. J Virol 82:7467–7474.
