# Supplementary material for: Genotypic and Phenotypic Diversity of Herpes Simplex Virus 2 within the Infected Neonatal Population
Source: mSphere. 2019 Feb 27;4(1):e00590-18. doi: 10.1128/mSphere.00590-18 (PMC6393728; doi:10.1128/mSphere.00590-18)
Supplement: TEXT S1 [file mSphere.00590-18-s0001.pdf]

Supplemental Information for

Genotypic and phenotypic diversity within the neonatal HSV-2 population

Lisa N. Akhtar, Christopher D. Bowen, Daniel W. Renner, Utsav Pandey[2], Ashley N. Della Fera, David W. Kimberlin, Mark N. Prichard, Richard J. Whitley, Matthew D. Weitzman*, Moriah L. Szpara*

***Co-Corresponding Authors**
Moriah L. Szpara, moriah@psu.edu
Matthew D. Weitzman, weitzmanm@email.chop.edu

Virus source

Viruses analyzed were collected from neonates with HSV-2 infection, enrolled in three published clinical studies (1–3) by the Collaborative Antiviral Study Group (CASG) at the University of Alabama Birmingham (UAB). These HSV isolates originated from patients with SEM, CNS, or disseminated (DISS) disease with CNS involvement (**Table 1**). Samples were collected at the time of diagnosis, prior to initiation of acyclovir therapy. CNS11 and DISS14 were isolated from the cerebrospinal fluid (CSF) and all other viruses were isolated from the skin. CSF was collected from all infants at the time of diagnosis. However, for all of the viruses isolated from the skin of infants with CNS disease (CNS03, CNS12, CNS15, CNS17, and DISS29), the CSF collected at the time of diagnosis was PCR positive but culture negative for HSV. Viral isolates are associated with de-identified clinical information including age, sex, race, and clinical morbidity scores, as approved by the UAB Institutional Review Board (IRB). Enrollment in the original studies was evenly split between males and females, and included both black and white patients. Clinical morbidity score was determined by the CASG after 12 months of life as (1)

normal; (2) mild impairment which includes ocular sequelae (keratoconjunctivitis), speech delay, or mild motor delay; (3) moderate impairment which includes hemiparesis, persistent seizure disorder, or developmental delay of less than or equal to 3 months adjusted developmental age; (4) severe impairment which includes microcephaly, spastic quadriplegia, chorioretinitis or blindness, or a serious developmental delay of least 3 months according to the Denver Developmental Assessment Scale (1, 2). These assessments were performed at each collaborating site. Initial collection of samples was approved by the UAB IRB. Use of these samples in this study was approved by the UAB IRB.

Cell culture

Human lung fibroblast MRC-5 cells (ATCC®, CCL-171) were cultured in minimum essential medium Eagle (MEME, Sigma-Aldrich; M5650) supplemented with 10% fetal bovine serum (FBS, Hyclone; SH30071.03), 2mM L-glutamine (Gibco, A2916801) and 1X penicillin-streptomycin (Gibco; 15140122). African green monkey kidney Vero cells (ATCC®, CCL-81) and human epithelial bone osteosarcoma U2OS cells (ATCC®, HTB-96) were cultured in Dulbecco's modified Eagle's medium with high glucose (DMEM, Hyclone; SH30081.02) supplemented with 10% FBS, 2mM L-glutamine, and 1X penicillin-streptomycin. Cell lines were authenticated by ATCC prior to purchase, and were confirmed to be mycoplasma free throughout experiments by periodic testing (LookOut Mycoplasma, Sigma).

Virus culture

Initial viral isolates (passage 1-2) were obtained from CASG samples stored at the UAB. Each isolate had been cultured at the time of diagnosis, aliquoted after one passage, and snap frozen.

To conduct viral genome sequencing and phenotypic assays, each stock was expanded over three serial passages by infecting monolayers of human MRC-5 cells at a MOI of 0.01. Each infection was allowed to progress to complete cytopathic effect (CPE) before harvest (between 50-70 hours), with increasing cell volume at each passage to produce sufficient virions for subsequent phenotypic and genomic comparisons. Viral stocks were titered on monolayers of either Vero cells or U2OS cells, for 100 or 48 hours respectively, to allow plaques to develop. Plaque formation was facilitated by limiting viral diffusion with a methylcellulose overlay. Plaque size and morphology were monitored carefully and did not change for any viral isolates over the course of virus stock expansion.

Plaque measurements

After appropriate incubation plaques were stained with 0.5% methylene blue and allowed to dry. Serial 4X brightfield images were collected on an EVOS FL Auto Imaging System and stitched by EVOS software to create an image of the entire well (University of Pennsylvania Cell and Developmental Biology Microscopy Core). No processing was performed. The area of 100 plaques was measured for each viral isolate using ImageJ software.

Genome copy number estimation by quantitative PCR for UL27

DNA was extracted using a PureLink genomic DNA mini kit (ThermoFisher Scientific). Viral genome copy number was determined using an established assay based on real-time PCR using primers and dual-fluorescent probe specific to viral glycoprotein B gene (gB; UL27) . Samples were assayed alongside a standard curve of HSV-1 strain F nucleocapsid DNA (3), on a ViiA 7 Real-Time PCR System.

Viral entry assay

Monolayers of Vero cells were cooled to 4°C for 30 minutes prior to infection and 100 PFU of each viral isolate was applied to cell monolayers at 4°C for 1 hour to allow virus binding, after which unbound virus was washed from the cells. Cells were then moved to 37°C to allow virus entry. At 0, 10, 20, 30, 45, or 60 minutes, a low-pH citrate buffer was applied to infected cells to inactivate virus that had not penetrated the cellular membrane. At each time point and for each virus, parallel infections were performed without the addition of citrate solution. These served as controls to determine the maximum number of plaques formed. Cell monolayers were washed and overlaid with methylcellulose. Plaques were scored after 100 hours of incubation. Viral entry was quantified as the fraction of plaques formed following citrate buffer application, where 100% is the number of plaques formed on a monolayer not treated with citrate buffer (control).

Single-step and multi-step growth curves

Virus diluted in basic growth media containing 2% FBS was applied to near confluent monolayers of Vero cells and allowed to adsorb for 1 hour. Virus was removed and cells were incubated at 37C for the duration of infection. At 2 hpi, media containing 0.1% human serum was added to reduce the contribution of cell-free spread of virus. Single-step growth curves were performed at MOI=5 as defined by titering viral stocks on U2OS cells, and monolayers harvested at 2, 6, 12, and 24 hpi. Multi-step growth curves were performed at MOI=0.001 as defined by titering viral stocks on U2OS cells, and monolayers were harvested at 2, 24, 48, and 72 hpi. Every 24 hours, the supernatant was removed and media containing 0.1% human serum reapplied. At the conclusion of each infection, cells were washed two times with PBS and

collected by scraping into an equal volume of media.

Immunocytochemistry

Multi-step growth curves were performed as described above in Vero cell monolayers grown on glass coverslips. To terminate each infection, coverslips were washed with PBS and fixed in 4% paraformaldehyde for 15 minutes at room temperature. Cells were permeabilized with 0.5% Triton-X, blocked in 3% BSA, and incubated with polyclonal rabbit antibodies raised against total HSV (Agilent Dako, B0114). After washing, cells were incubated with fluorophore-conjugated anti-Rabbit secondary antibodies (Invitrogen, A-11008) to mark HSV infected cells, and counterstained with DAPI to mark cell nuclei. Coverslips were initially visualized with a Leica DM6000 wide field microscope (UPenn Cell and Developmental Microscopy Core). The 5X images were collected on a Photometrics HQ2 high resolution monochrome CCD camera, and processed with LAS AF software. The 10X images were collected on a EVOS FL Auto Imaging System (UPenn Cell and Developmental Microscopy Core) and stitched using EVOS software. Exposure and gain were optimized within each experiment for one virus at the 72-hour time point and applied identically to each image within that experiment. Any subsequent image processing (ImageJ) was applied equally to all images in a given experiment.

Immunoblotting

Whole cell lysates were prepared with 1X LDS sample buffer (NuPage) and equal amounts of lysate were separated by SDS-PAGE. Membranes were immunoblotted with polyclonal rabbit antibodies raised against total HSV (Agilent Dako, B0114); glycoproteins (g)C, gD, gE, gH, and VP5 (all gifts from Gary Cohen); ICP8 (gift from David Knipe); and GAPDH (GeneTex,

GTX100118). Proteins were visualized with Pierce ECL Western Blotting Substrate (ThermoFisher Scientific) and detected using a G:Box imaging system (Syngene).

Viral DNA isolation and Illumina sequencing

Viral nucleocapsid DNA for genome sequencing was prepared by infecting MRC-5 cells at an MOI ≥5 as previously described (4, 5). Viral nucleocapsid gDNA was sheared using a Covaris M220 sonicator/disruptor under the following conditions: 60s duration, peak power 50, 10% duty cycle, at 4°C. Barcoded sequencing libraries were prepared using the Illumina TruSeq low-throughput protocol according to manufacturer's specifications and as previously described (6, 7). The quality of sequencing libraries was evaluated by Qubit (Invitrogen, CA), Bioanalyzer (Agilent), and qPCR (KAPA Biosystems). Paired-end sequencing (2 × 300bp length) was performed on an Illumina MiSeq, according to manufacturer's recommendations (17pM input).

De novo genome assembly

A consensus genome was assembled for each viral isolate using a previously described Viral Genome Assembly (VirGA) bioinformatics workflow (6). VirGA begins by quality-filtering the MiSeq sequence reads and removing sequences that match the host (human) genome. Thereafter VirGA uses a combination of SSAKE *de novo* assemblies run with differing parameters, which are then combined into a single draft genome using Celera and GapFiller (8–10). After quality-checking and iterative improvement of the genome assembly, annotations were transferred from the HSV-2 reference genome (strain HG52; GenBank NC_001798) to each new genome based on sequence homology (11).

Comparative genomics and phylogenetic analysis

The genomes of all 10 neonatal HSV-2 isolates were combined with all annotated HSV-2 genomes available in GenBank (see **Table S1** for full list) and aligned using MAFFT (12). All previously published HSV-2 genomes were derived from infected adults. The genome-wide alignment used a trimmed genome format (lacking the terminal repeats) to avoid giving undue weight to these duplicated sequences. The MAFFT alignment was used to generate a NeighborNet phylogenetic network in SplitsTree with Uncorrected P distances (13–15). A diverse subset of ten adult HSV-2 isolates was selected for protein-level comparisons with the ten neonatal isolates; these are indicated in **Table S1** with an asterisk. GenBank genomes that lacked open reading frame (ORF) and protein annotations were excluded from all comparisons (16, 17). ClustalW2 was used to construct pairwise nucleotide alignments between whole genomes and pairwise amino acid alignments for each gene and protein (18). Pan-HSV-2 comparisons excluded three viral proteins for which sequences are not fully determined in most published strains, likely due to the high G+C-content and numerous tandem repeats in these regions: ICP34.5 (RL1 – annotated/complete in only 9 genomes from Table S1) and ICP4 (RS1 – annotated/complete in only 6 genomes from Table S1), ICP0 (RL2 – annotated/complete in only 8 genomes from Table S1) (16, 19, 20). Custom Python scripts were used on these alignments to identify nucleotide and AA differences between samples.

References for Supplemental Methods

1. Whitley R, Arvin A, Prober C, Burchett S, Corey L, Powell D, Plotkin S, Starr S, Alford C, Connor J, Jacobs R, Nahmias A, Soong S-J, the National Institute of Allergy, Infectious Diseases Collaborative Antiviral Study Group*. 1991. A Controlled Trial Comparing Vidarabine with Acyclovir in Neonatal Herpes Simplex Virus Infection. N Engl J Med 324:444–449.

2. Whitley R, Arvin A, Prober C, Corey L, Burchett S, Plotkin S, Starr S, Jacobs R, Powell D, Nahmias A, Sumaya C, Edwards K, Alford C, Caddell G, Soong S-J, the National Institute of Allergy and Infectious Diseases Collaborative Antiviral Study Group*. 1991. Predictors of Morbidity and Mortality in Neonates with Herpes Simplex Virus Infections. N Engl J Med 324:450–454.

3. Szpara ML, Parsons L, Enquist LW. 2010. Sequence variability in clinical and laboratory isolates of herpes simplex virus 1 reveals new mutations. J Virol 84:5303–13.

4. Szpara ML, Tafuri YR, Enquist LW. 2011. Preparation of viral DNA from nucleocapsids. J Vis Exp JoVE 2–7.

5. Szpara M. 2014. Isolation of herpes simplex virus nucleocapsid DNA, p. 31–41. *In* Diefenbach, RJ, Fraefel, C (eds.), Herpes Simplex Virus. Springer New York, New York, NY.

6. Parsons LR, Tafuri YR, Shreve JT, Bowen CD, Shipley MM, Enquist LW, Szpara ML. 2015. Rapid genome assembly and comparison decode intrastrain variation in human alphaherpesviruses. mBio 6:e02213-14.

7. Bowen CD, Renner DW, Shreve JT, Tafuri Y, Payne KM, Dix RD, Kinchington PR, Gatherer D, Szpara ML. 2016. Viral forensic genomics reveals the relatedness of classic herpes simplex virus strains KOS, KOS63, and KOS79. Virology 492:179–186.

8. Myers EW, Sutton G, Delcher AL, Dew IM, Fasulo DP, Flanigan MJ, Kravitz SA, Mobarry CM, Reinert KHJ, Remington KA, Anson EL, Bolanos RA, Chou H-H, Jordan CM, Halpern AL, Lonardi S, Beasley EM, Brandon RC, Chen L, Dunn PJ, Lai Z, Liang Y, Nusskern DR, Zhan M, Zhang Q, Zheng X, Rubin GM, Adams MD, Venter CJ. 2000. A Whole-Genome Assembly of Drosophila. Science 287:2196–2204.

9. Boetzer M, Pirovano W. 2012. Toward almost closed genomes with GapFiller. Genome Biol 13:1–9.

10. Warren RL, Sutton GG, Jones SJM, Holt R a. 2007. Assembling millions of short DNA sequences using SSAKE. Bioinformatics 23:500–1.

11. Dolan A, Jamieson FE, Cunningham C, Barnett BC, McGeoch DJ. 1998. The genome sequence of herpes simplex virus type 2. J Virol 72:2010–2021.

12. Katoh K, Misawa K, Kuma K, Miyata T. 2002. MAFFT: a novel method for rapid multiple sequence alignment based on fast Fourier transform. Nucleic Acids Res 30:3059–3066.

13. Strimmer K, Wiuf C, Moulton V. 2001. Recombination analysis using directed graphical models. Mol Biol Evol 18:97–99.

14. Norberg P, Kasubi MJ, Haarr L, Bergstrom T, Liljeqvist J-A. 2007. Divergence and recombination of clinical herpes simplex virus type 2 isolates. J Virol 81:13158–13167.

15. Norberg P, Tyler S, Severini A, Whitley R, Liljeqvist J-A, Bergstrom T. 2011. A genome-wide comparative evolutionary analysis of herpes simplex virus type 1 and varicella zoster virus. PloS One 6:1–8.

16. Newman RM, Lamers SL, Weiner B, Ray SC, Colgrove RC, Diaz F, Jing L, Wang K, Saif S, Young S, Henn M, Laeyendecker O, Tobian AAR, Cohen JI, Koelle DM, Quinn TC, Knipe DM. 2015. Genome Sequencing and Analysis of Geographically Diverse Clinical Isolates of Herpes Simplex Virus 2. J Virol JVI.01303-15.

17. Johnston C, Magaret A, Roychoudhury P, Greninger AL, Reeves D, Schiffer J, Jerome KR, Sather C, Diem K, Lingappa JR, Celum C, Koelle DM, Wald A. 2017. Dual-strain genital herpes simplex virus type 2 (HSV-2) infection in the US, Peru, and 8 countries in sub-Saharan Africa: A nested cross-sectional viral genotyping study. PLOS Med 14:e1002475.

18. Larkin MA, Blackshields G, Brown NP, Chenna R, McGettigan PA, McWilliam H, Valentin F, Wallace IM, Wilm A, Lopez R, Thompson JD, Gibson TJ, Higgins DG. 2007. Clustal W and Clustal X version 2.0. Bioinforma Appl Note 23:2947–2948.

19. Kolb AW, Larsen IV, Cuellar JA, Brandt CR. 2015. Genomic, Phylogenetic, and Recombinational Characterization of Herpes Simplex Virus 2 Strains. J Virol 89:6427–6434.

20. Johnston C, Magaret A, Roychoudhury P, Greninger AL, Cheng A, Diem K, Fitzgibbon MP, Huang M, Selke S, Lingappa JR, Celum C, Jerome KR, Wald A, Koelle DM. 2017. Highly conserved intragenic HSV-2 sequences: Results from next-generation sequencing of HSV-2 U L and U S regions from genital swabs collected from 3 continents. Virology 510:90–98.
